# HDAC1-mediated SUCLA2 lysine β-hydroxybutyrylation modulates myocardial mitochondrial function in sepsis-induced heart dysfunction

**DOI:** 10.1016/j.redox.2026.104431

**Published:** 2026-10-08

**Authors:** Ri Wen, Wanshan Ning, Xin-Mei Huang, Peng-Hui Hao, Jingjing Yang, Wei Liu, Yu-Hang Yang, Chun-Feng Liu, Ni Yang, Yaping Guo, Tie-Ning Zhang

**Affiliations:** a Department of Pediatrics, PICU, Shengjing Hospital of China Medical University, Shenyang, 110004, China; b Institute for Clinical Medical Research, The First Affiliated Hospital of Xiamen University, School of Medicine, Xiamen University, Xiamen, 361003, China; c Department of Endocrinology, Shanghai Fifth People's Hospital, Fudan University, Shanghai, 200240, China; d Department of Pulmonary and Critical Care Medicine, The First Affiliated Hospital of Xiamen University, School of Medicine, Xiamen University, Xiamen, 361003, China; e Department of Pathophysiology, School of Basic Medical Sciences, Zhengzhou University, Zhengzhou, 450001, China

**Keywords:** Sepsis-induced heart dysfunction, Mitochondrial function, Lysine β-hydroxybutyrylation, Succinate-CoA ligase ADP-Forming subunit beta, Histone deacetylase 1

## Abstract

**Background:**

Lysine β-hydroxybutyrylation (Kbhb) is associated with energy regulation and mitochondrial function, and its role in sepsis-induced heart dysfunction has not been elucidated.

**Objectives:**

Reveals the extensive effects of Kbhb in sepsis-induced heart dysfunction and identifies the potential targets.

**Methods:**

A septic heart dysfunction rat model was induced by lipopolysaccharide intraperitoneal injection. Proteomic and β-hydroxybutyrylomic analyses were conducted to assess alterations in Kbhb between cardiac tissues from the control and lipopolysaccharide rats. Putative regulatory proteins were identified by leveraging a multifaceted hybrid learning framework combined with protein language models. Lysine to arginine (R) mutation was made to mimic decreased Kbhb levels. Transcriptomic analysis was further performed to predict the potential downstream mechanisms.

**Results:**

Succinate-CoA Ligase ADP-Forming Subunit Beta (SUCLA2) was selected because of its role in mitochondrial function, and the Kbhb level at the K444 site was significantly different. Decreased Kbhb level at K444 of SUCLA2 was confirmed in septic cardiac tissues and lipopolysaccharide-treated H9c2 cardiomyocytes using specific antibody. SUCLA2^K444R^ mutant reversed the protective effects of SUCLA2^WT^ on mitochondrial function in lipopolysaccharide-treated H9c2 cardiomyocytes, accompanied by decreased succinyl-CoA synthetase activity, reduced mitochondrial membrane potential, suppressed adenosine triphosphate production, and increased reactive oxygen species accumulation. The aggravating effects of the SUCLA2^K444R^ mutant on septic heart dysfunction were confirmed *in vivo* using the specific promoter. Mechanistically, histone deacetylase 1 inhibited SUCLA2 Kbhb at K444 residue.

**Conclusions:**

Reduced SUCLA2 Kbhb level at K444 site aggravates myocardial mitochondrial dysfunction, leading to sepsis-induced heart dysfunction. This study reveals a novel function of Kbhb in sepsis-induced heart dysfunction and elucidates the mechanism of SUCLA2 K444 Kbhb.

## Highlights

• Multi-omics analyses show reduced SUCLA2 Kbhb level in sepsis-induced heart dysfunction.
• FuncKbhb predicts the K444 residue might serve as functional site of SUCLA2.
• Reduced Kbhb level at K444 residue of SUCLA2 mediates mitochondrial dysfunction in cardiomyocytes.
• HDAC1 interacts with and mediates the Kbhb modification of SUCLA2 at K444 residue.
• SUCLA2^K444R^ mutant in cardiomyocytes exacerbates sepsis-induced heart dysfunction in rats.

## Graphical abstract

**Figure:**
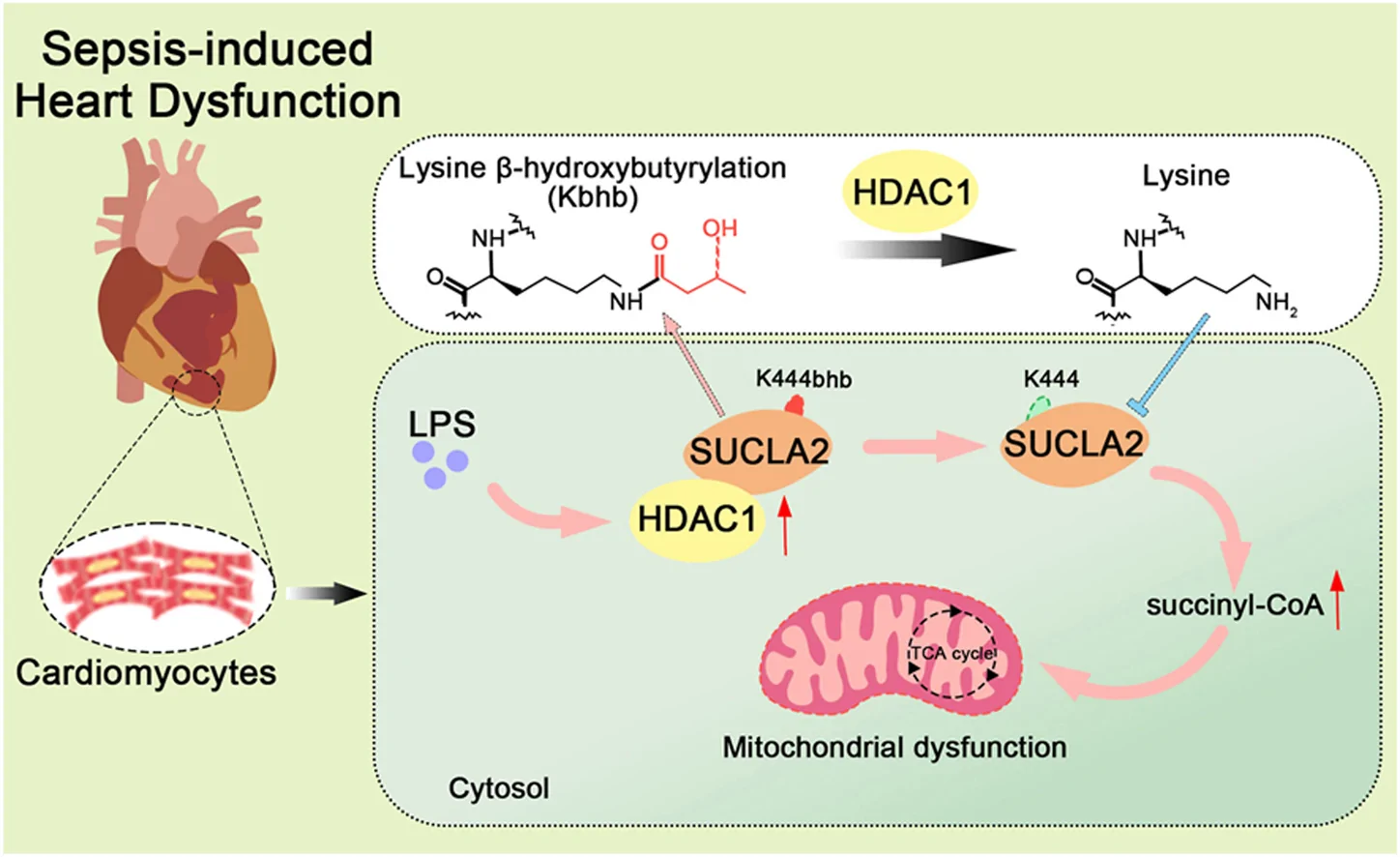

## 1 Introduction

Sepsis is a complex illness resulting from an altered systemic host response to infection, causing physiological, pathological, and biochemical abnormalities [1]. This exaggerated immune response leads to widespread tissue damage, organ dysfunction, and potentially severe complications, including mortality [2]. Sepsis-induced heart dysfunction, also referred to as sepsis-induced myocardial depression, is characterized by impaired myocardial contractility and a reduced ejection fraction [3,4]. This phenomenon is associated with elevated mortality rates, particularly among patients who are experiencing septic shock. Thus, elucidating the pathogenesis of septic cardiac dysfunction and investigating targeted therapeutic approaches will positively influence the outcomes of patients suffering from sepsis. Post-translational modifications, such as acetylation [5], succinylation [6], and sulfonylation [7], have been verified to modulate sepsis-induced heart dysfunction. Lysine β-hydroxybutyrylation (Kbhb) is a novel modification that entails the covalent attachment of a β-hydroxybutyryl group (structural formula: –CO–CH_2_–CH(OH)–CH_3_) to the ε-amino group of a lysine residue, thereby forming an amide bond [8]. Kbhb reshapes protein function through various molecular mechanisms. The Kbhb modification of histone proteins regulates chromatin structure and downstream gene transcription [9,10]. The Kbhb of non-histone proteins, on the other hand, has been shown to directly regulate enzyme activity and protein stability [11]. Kbhb exhibits broad functional significance as it contributes to the progression of various diseases such as cancer [12], alcoholic liver disease [13], and acute liver injury [14]. The progression of colorectal cancer is promoted by the reprogramming of arginine metabolism, driven by abnormal Kbhb modification of ARG1 [15]. The Kbhb modification at OXCT1 K421 enhances its activity, thereby accelerating the utilization of ketone and ultimately maintaining ketone body metabolism in starvation, ketosis, or type 1 diabetes [16]. Above studies demonstrated that proteins with Kbhb modification mediate metabolic processes and thereby influence disease progression.

Mitochondria serve as the primary energy powerhouses of cardiomyocytes, generating cellular adenosine triphosphate (ATP) to sustain continuous cardiac contractility, while its dysfunction has been suggested to drive sepsis-induced myocardial dysfunction [17]. Previous study suggested that an increased level of SNX9 Kbhb attenuates alcohol-induced liver damage by enhancing mitochondrial activity [18]. Therefore, we aimed to investigate the potential functions of Kbhb and the modification sites of functional proteins in sepsis-induced heart dysfunction.

In this study, the lipopolysaccharide-induced rat model of sepsis-induced heart dysfunction was established [19]. The characteristics of Kbhb modification in sepsis-induced heart dysfunction were determined using proteomic and β-hydroxybutyrylomic profiling. Furthermore, we established a functional Kbhb model (FuncKbhb), a hybrid protein language model (PLM)–based framework that integrates multifeatured learning and transfer learning to predict functional Kbhb sites. Our findings might serve as the experimental foundation for the functional roles of Kbhb and its contribution to sepsis-induced heart dysfunction.

## 2 Methods

### 2.1 Sepsis rat model

The *in vivo* procedures were conducted in accordance with the ARRIVE guidelines and the National Institutes of Health Guide for the Care and Use of Laboratory Animals. This study received approval from the Institutional Animal Care and Use Committee of Shengjing Hospital of China Medical University. Male Sprague–Dawley rats (6 weeks old) were provided autoclaved rat chow with free access to water, and housed in a temperature-controlled environment maintained at 22 ± 1°C with a 12-h light/dark cycle. The septic shock model was induced by intraperitoneal injection of lipopolysaccharide (20 mg/kg), as described previously [20]. The rats were humanely euthanized with CO_2_ after the lipopolysaccharide injection, and the heart tissue was harvested.

### 2.2 Proteomic and β-hydroxybutyrylomic profiling

The proteomic and β-hydroxybutyrylomic profiling was performed by Hangzhou Jingjie Biotechnology Co., Ltd. The specific information is provided in the supplementary materials.

### 2.3 Cell culture and treatment

H9c2 cardiomyocytes were maintained in Dulbecco's modified Eagle's medium (DMEM, Servicebio, China) supplemented with 10% fetal bovine serum (FBS, Tianhang, China). The plasmid containing the 3✕ Flag tag and the arginine mutation at position K444 of the SUCLA2 gene (SUCLA2^K444R^), or the wild-type SUCLA2 gene containing the 3✕ Flag tag (SUCLA2^WT^), was transiently transfected into cells using Lipofectamine 3000 (Invitrogen, USA). After 48 h, the H9c2 cardiomyocytes were treated with 10 μg/mL lipopolysaccharide for 12 h.

### 2.4 *In vivo* experiments of SUCLA2 mutation in cardiomyocytes

The arginine mutation at position K444 of the SUCLA2 gene (SUCLA2^K444R^) was used to mimic an unmodified lysine that cannot be β-hydroxybutyrylated. An adeno-associated virus type 9 (AAV9) system using the cardiac troponin T (cTnT) promoter was selected for the delivery of cardiomyocyte-specific genes [21]. Recombinant AAV9-cTnT-Vector (AAV9^cTnT^-Vector), AAV9-cTnT-SUCLA2-WT-Flag (AAV9^cTnT^-SUCLA2^WT^-Flag), and AAV9-cTnT-SUCLA2-K444R mutation-Flag (AAV9^cTnT^-SUCLA2^K444R^-Flag) viruses were constructed. Two weeks before lipopolysaccharide injection, the viruses were injected via the tail vein.

### 2.5 Echocardiographic examinations

Echocardiography was conducted to assess cardiac function before sacrifice. The rats were anesthetized with isoflurane (3% for induction and 2% for maintenance). Rats were placed in a supine position, and the body temperature was maintained. The Vinno 6th ultrasound imaging system was used for echocardiography measurements.

### 2.6 Transmission electron microscopy of mitochondria

Rat myocardial tissues were quickly excised and immediately preserved in a 0.1 M phosphate buffer containing 1% osmium tetroxide for 2 h. The tissues were dehydrated, embedded, and sectioned into 60- to 80-nm slices. The sections were stained for 8 min with 2% uranyl acetate in pure ethanol in the dark, followed by 8 min in 2.6% lead citrate solution without CO_2_. Images were obtained by transmission electron microscopy (H-7650, Japan).

### 2.7 Immunoprecipitation‐liquid chromatography‐mass spectrometry (IP-LC/MS)

Proteins were extracted from lipopolysaccharide-treated H9c2 cardiomyocytes for further IP-LC/MS analysis. The eluent containing SUCLA2-interacting proteins was loaded onto a sodium dodecyl sulfate–polyacrylamide gel. Following staining with Coomassie blue, the target bands were excised and processed. LC-MS analysis was performed to identify protein–protein interactions. Peptide sequences (and thus protein identities) were determined by matching acquired fragmentation patterns with protein databases.

### 2.8 Transcriptomic analysis

Total RNA from SUCLA2^K444R^ H9c2 cells and SUCLA2^WT^ H9c2 cells under lipopolysaccharide were extracted. Briefly, after the RNA samples pass quality control, the eukaryotic mRNA is enriched using magnetic beads coated with oligo(dT). The fragmentation buffer is introduced to cleave the mRNA into short fragments. Utilizing the mRNA as a template, single-stranded cDNA is synthesized with random hexamers. Following this, buffer, dNTPs, DNA polymerase I, and RNase H are added to synthesize double-stranded cDNA, which is then purified using AMPure XP beads. The purified double-stranded cDNA undergoes end repair, A-tailing, and the incorporation of sequencing adapters, followed by size selection with AMPure XP beads. Finally, PCR amplification is conducted, and the PCR products are purified using AMPure XP beads to yield the final sequencing library using the Illumina platform. The differentially expressed genes were generated using a cutoff value of fold change >1.5 or <0.667 and *p*. value < 0.05. The differentially expressed genes were then subjected to GO functional enrichment and KEGG pathway analyses. GO terms and KEGG pathways that met the criterion of a p-value < 0.05 were identified.

### 2.9 Statistical analysis

Statistical evaluations were conducted using GraphPad Prism statistical software. *P* < 0.05 was considered statistically significant. Comparisons between different groups were made using either t-tests, one-way ANOVA, or two-way ANOVA.

## 3 Results

### 3.1 Proteome and β-hydroxybutyrylome alterations in sepsis-induced heart dysfunction

Lysine β-hydroxybutyrylation (Kbhb) is a novel acylation modification of proteins mediated by β-hydroxybutyrate that associates with energy metabolism regulation [22]. Abnormal energy metabolism is the key mechanisms in septic cardiomyopathy, yet the role of Kbhb in this condition remains poorly characterized. Myocardial tissue samples were collected from the control and lipopolysaccharide-induced septic heart dysfunction rats for proteomic and β-hydroxybutyrylomic profiling (Fig. 1A). We checked the raw data of detected peptides (Fig. S1E), and median relative standard deviation values meet the established criteria (Fig. S1C and S1F). The raw proteomic data and the modified peptides detected and found the majority consistent with higher-energy collisional dissociation fragmentation (Fig. S1A and S1D). A total of 3166 proteins were traced and supported by ≥ two peptides (Fig. S1B). A total of 1220 proteins (Table S1) and 2667 Kbhb sites were identified (Fig. 1B and C, and Table S2). Up to 2618 Kbhb sites had a localization probability (LP) score of one (Fig. 1D). Overall, our quantification results are robust and highly reliable at both the protein and site levels. We compared the two groups of samples and found that there were more Kbhb sites in septic rat myocardial tissue (Fig. 1E). To investigate the Kbhb motifs, we further analyzed the amino acid preferences surrounding Kbhb sites and found that phenylalanine and leucine residues were highly enriched at the +1 position (Fig. 1F). To comprehensively characterize proteome and Kbhb alterations in sepsis-induced heart dysfunction, we identified 69 differentially expressed Kbhb sites and 40 differentially expressed proteins, revealing key molecular changes underlying the condition (Fig. 1G and H, Table S3, and Table S4). Notably, although many Kbhb sites were significantly changed in the sepsis group, the overall expression level of their corresponding proteins did not change significantly (Fig. 1I).

**Fig. 1:**
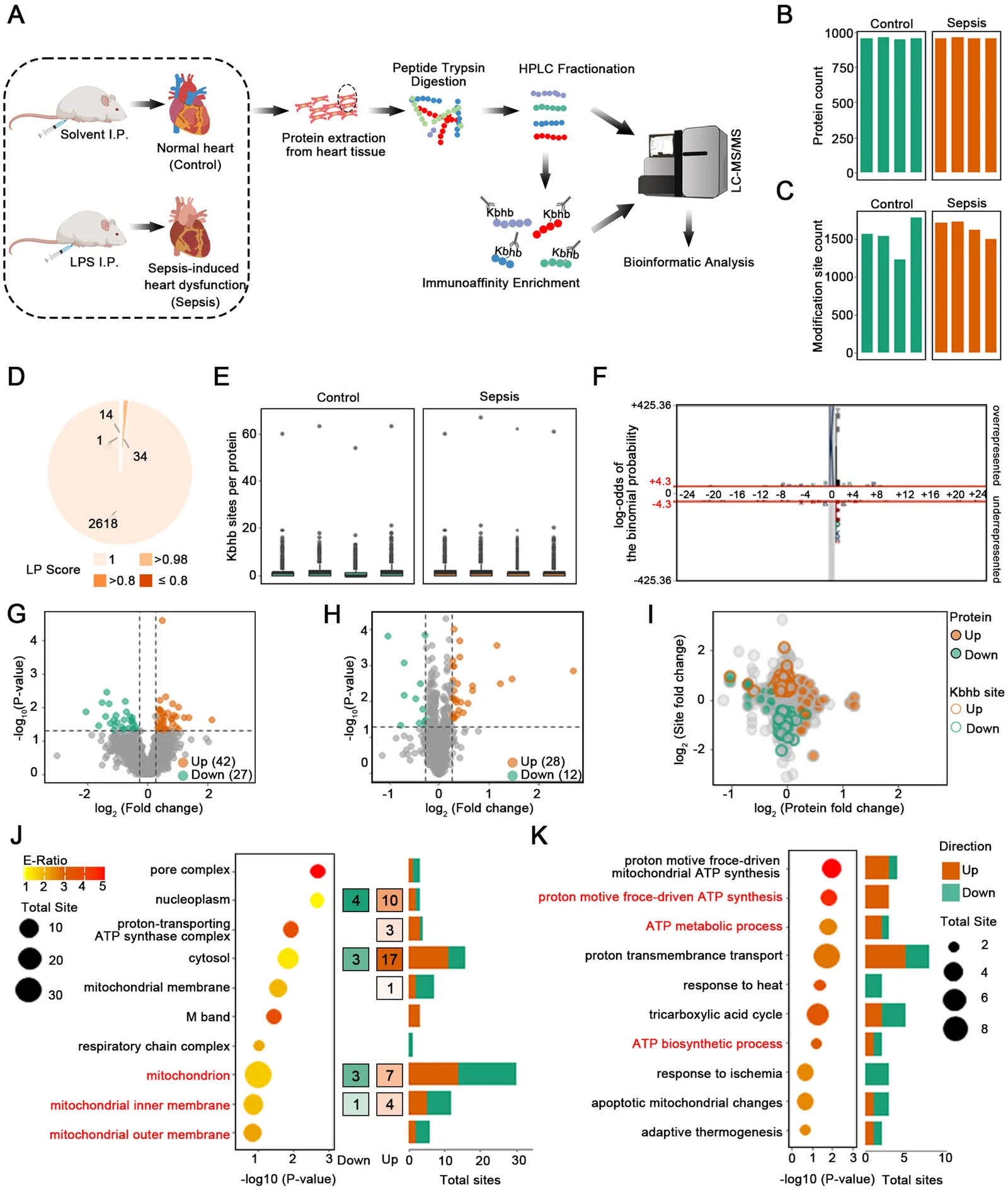
**Proteome and lysine β-hydroxybutyrylome alterations in sepsis-induced heart dysfunction** (A) Experimental workflow for proteomic and β-hydroxybutyrylomic analysis in septic cardiomyopathy rat models. Myocardial tissues from control and sepsis-induced heart dysfunction groups were subjected to protein extraction, tryptic digestion, HPLC fractionation, immunoaffinity enrichment of Kbhb peptides, and LC-MS/MS analysis. (B, C) Distribution of quantified proteins (B) and Kbhb sites (C) in control and sepsis-induced heart dysfunction groups (biological replicates: N = 4). (D) LP score distribution of identified Kbhb sites. (E) Boxplot showing the distribution of Kbhb sites numbers per protein across samples, with discrete points displayed (biological replicates: N = 4). (F) Sequence motif analysis showing amino acid preferences surrounding identified Kbhb sites. (G, H) Volcano plots displaying differential Kbhb sites (G) and proteins (H) between sepsis-induced heart dysfunction and control groups (FC > 1.2 or FC < 0.83, P < 0.05, biological replicates: N = 4) (I) Scatter plot correlating Kbhb site-level alterations with corresponding protein expression changes (biological replicates: N = 4). (J, K) GO enrichment analysis of cellular components (J) and biological processes (K) for proteins harboring differential Kbhb sites (two-sided hypergeometric test, m > 3, P < 0.05). Heatmap values indicate numbers of up-/down-regulated β-hydroxybutyrylated proteins; bar charts show corresponding Kbhb site counts. E-ratio scale: 1-5 for both panels.

GO enrichment analysis on proteins containing these sites was further performed to explore the functional implications of differential Kbhb modification (Fig. 1J and K, and Table S5). Proteins with Kbhb modification were significantly enriched in mitochondrial energy metabolism pathways and stress response processes, suggesting that Kbhb may contribute to cellular adaptation to environmental stress and maintenance of energy homeostasis. Kbhb-modified proteins were mainly localized to mitochondrial structures (Fig. 1J and K). Taken together, these findings suggested that Kbhb functions in sepsis-induced heart dysfunction via mitochondrial function and energy metabolism.

### 3.2 Development of FuncKbhb for the prediction of functional Kbhb sites

Despite advances in LC–MS/MS enabling more comprehensive identification of Kbhb sites, their functional roles are still poorly understood. Predicting functional Kbhb sites in all differentially expressed Kbhb sites (Fig. 2A) is crucial for understanding this post-translational modification, thereby illustrating its role in sepsis-induced heart dysfunction. Therefore, we developed FuncKbhb, a hybrid computational framework that integrates a protein language model (ESM2) with multi-feature learning to predict functional Kbhb sites, effectively tackling challenges such as limited labeled data and class imbalance (Fig. S1G). A functional Kbhb site is defined as a β-hydroxybutyrylated lysine residue capable of altering protein function, enzymatic activity or biological phenotypes. The design of FuncKbhb was inspired by widely-adopted strategies for predicting functionally important sites [23]. Our approach combines ESM2 embeddings with six complementary feature sets and employs a two-stage training strategy: pre-training on a large-scale Kbhb dataset containing 6197 Kbhb sites (Table S6) from literature, the CPLM database [24], and our experimental data to establish a base model, followed by fine-tuning using a small set of functional Kbhb sites (Table S7) via transfer learning. The two-stage training strategy was designed to allow FuncKbhb to differentiate functionally impactful Kbhb sites from other non-functional β-hydroxybutyrylation events. For each modification site, a 51-residue sequence window centered on the modified lysine (25 amino acids upstream and downstream) was extracted as input. To mitigate data imbalance, we applied the synthetic minority over-sampling technique (SMOTE). During cross-validation, the pre-training phase achieved an AUC of 0.871 (Fig. 2B). The integration of ESM2 with multiple features enhanced model performance, increasing the AUC by 2.4% compared to models using individual features. For functional Kbhb site prediction, FuncKbhb attained an AUC of 0.838, surpassing single-feature models by 12.7% (Fig. 2C), demonstrating its superior predictive capability. With a prediction value threshold of 0.5, the confusion matrices showed that our models accurately identified Kbhb and functional Kbhb sites (Fig. 2D). Subsequently, FuncKbhb was used to score all differentially Kbhb-modified lysine sites. The top five sites were selected as candidate functional sites (Fig. 2E). SUCLA2 encodes the beta subunit of the ADP-forming enzyme, succinyl-CoA ligase, which catalyzes the reversible conversion of succinyl-CoA to succinate in the tricarboxylic acid cycle and promotes ATP production [25]. CANX encodes calnexin, an endoplasmic reticulum–resident chaperone that governs mitochondrial bioenergetics [26]. ATP1A2 maintains plasma membrane electrochemical gradients and is essential for the excitability and contractility of cardiomyocytes [27]. MRPS22 encodes a small-subunit protein of mitochondrial ribosomes, essential for mitochondrial translation [28]. KTN1 encodes kinectin 1, an endoplasmic reticulum integral membrane protein that acts as a major kinesin-binding partner and mediates the microtubule-dependent transport of membranous organelles, including the ER network [29]. The proteins harboring these prioritized sites are mainly involved in mitochondrial energy metabolism, which is linked to cardiac dysfunction [30]. Based on a comprehensive literature review, we identified key regulatory proteins that have been reported to be associated with mitochondrial dysfunction (Fig. 2F). SUCLA2 has been reported to be involved in diseases associated with mitochondrial metabolic abnormalities [25,31,32]. The identification spectra for the K444 modification site of the SUCLA2 protein were shown inFig. 2G. Sequence analysis of the SUCLA2 protein revealed homologous sequences near these amino acid residues in humans, mice, and rats (Fig. 2H and I). The high conservation of the K444 site of SUCLA2 among different species suggests that it may represent a functionally critical region. We hypothesized that Kbhb of SUCLA2 at the K444 site might regulate mitochondrial function in sepsis-induced heart dysfunction.

**Fig. 2:**
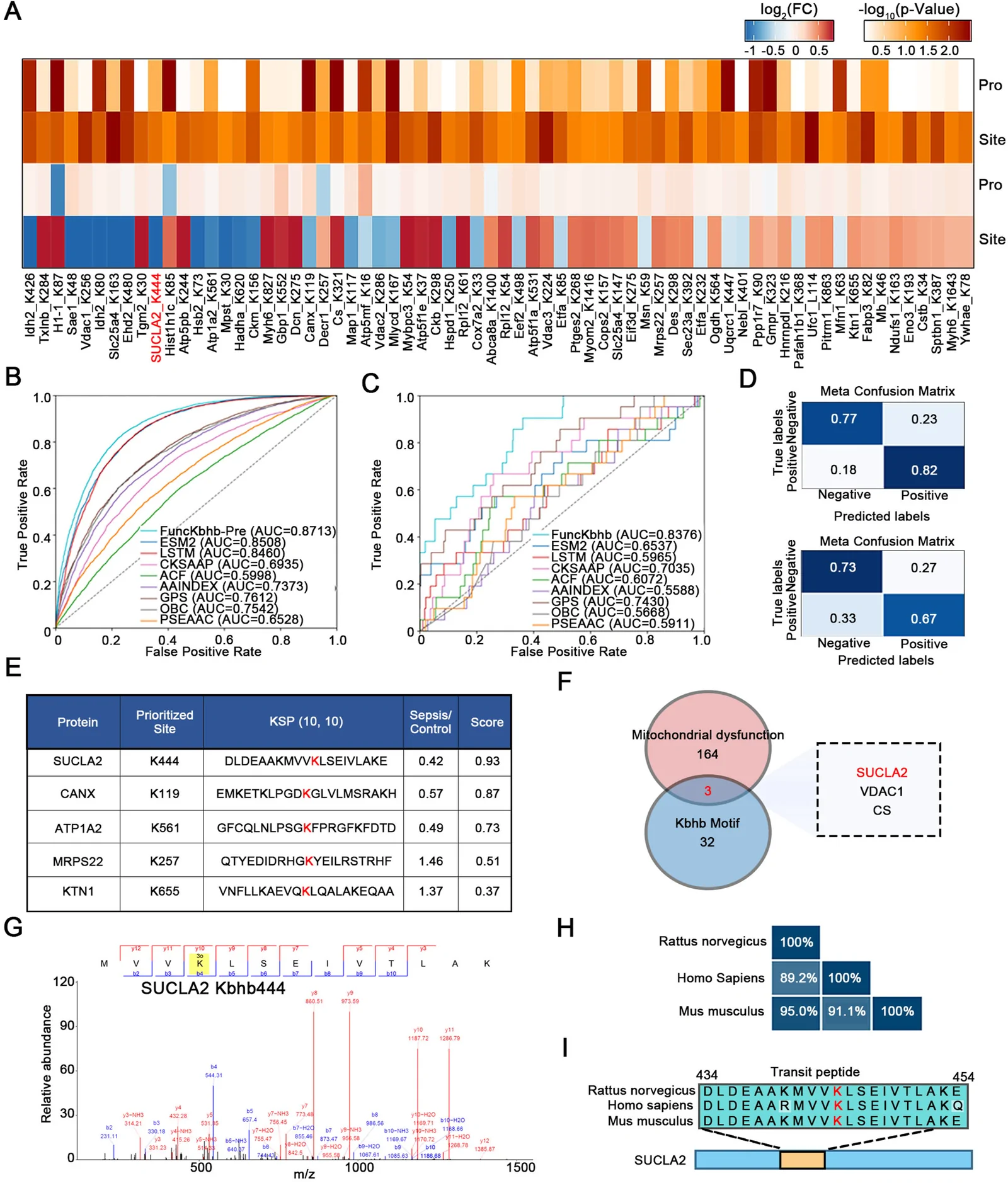
**Performance evaluation and comparison of FuncKbhb models and identified SUCLA2** (A) Differential expression analysis of Kbhb sites and corresponding proteins associated with adjusted P values and log_2_ (FC) (highlighting key differentially modified Kbhb of SUCLA2 at K444). (B) ROC curve of pre-trained models with different features for Kbhb site prediction (AUC values of 0.8713). (C) Performance comparison of different models for functional Kbhb site prediction (AUC values of 0.8376). (D) Confusion matrix for Kbhb site and functional Kbhb site prediction. (E) Functional prioritization of differentially Kbhb-modified lysine sites. Five sites were identified as functionally important. (F) Overlap between mitochondrial dysfunction-related proteins and Kbhb motif-containing proteins. (G) Identification mass spectra of the SUCLA2 Kbhb modification sites (H, I) Multiple sequence alignment of SUCLA2 across species (H) and conserved KSP regions (I).

Anti-β-Hydroxybutyryl-SUCLA2 (K444) Rabbit pAb was generated through a custom peptide-based immunization strategy. Two modified peptides (SUCLA2 K444bhb peptides A and B) were synthesized and validated by mass spectrometry to confirm sequence accuracy (Fig. S2A). Rabbits R3 and R4, immunized with peptide B, exhibited high titers and specificity for the modified peptide and were selected for further processing (Fig. S2B). ELISA results demonstrated that Ab3 recognized the modified SUCLA2 K444bhb peptide with a high titer (Fig. S2C). Dot blot analysis further confirmed its high specificity (Fig. S2D) and Western blot analysis revealed the expected molecular weight of approximately 50 kDa, corresponding to SUCLA2 (Fig. S2E). The septic rat model was established to validate the omics findings through the specificity antibodies. Echocardiographic results revealed disrupted cardiac function (Fig. 3A–C). There was a significant increase in serum levels of Creatine Kinase, MB Form (CK-MB) and Cardiac Troponin I (cTnI), indicating severe structural damage to cardiomyocytes in rats treated with lipopolysaccharide. The markedly elevated levels of serum Brain Natriuretic Peptide (BNP) suggest that the lipopolysaccharide injection induced cardiac dysfunction (Fig. 3D). H&E staining revealed extensive damage to the myocardial tissue in the lipopolysaccharide group, accompanied by extensive mitochondrial damage, as observed by transmission electron microscope (Fig. 3E). Injection of lipopolysaccharide resulted in inhibition of ATP generation and superoxide dismutase (SOD) activity. This is consistent with increased malondialdehyde (MDA) levels, suggesting aggravated oxidative damage in rat myocardial tissues (Fig. 3F). DHE staining also revealed an elevated level of reactive oxygen species (ROS) in the myocardial tissue of the rats (Fig. 3G). After verifying that the model of sepsis-induced heart dysfunction following lipopolysaccharide injection was successful, we validated the omics results. With the use of commercial pan-Kbhb antibody, we validated reduced SUCLA2 pan-Kbhb levels in lipopolysaccharide-treated rat myocardial tissue via coimmunoprecipitation experiment (Fig. 3H). In myocardial tissues of lipopolysaccharide treated rats, the protein expression of SUCLA2 showed no significant difference. The decreased Kbhb level of SUCLA2 at K444 was also observed in the myocardial tissue of septic heart dysfunction rats (Fig. 3I). Above all, we found that, in the myocardial tissue of rats with sepsis-induced heart dysfunction, Kbhb levels at the K444 site of SUCLA2 were reduced.

**Fig. 3:**
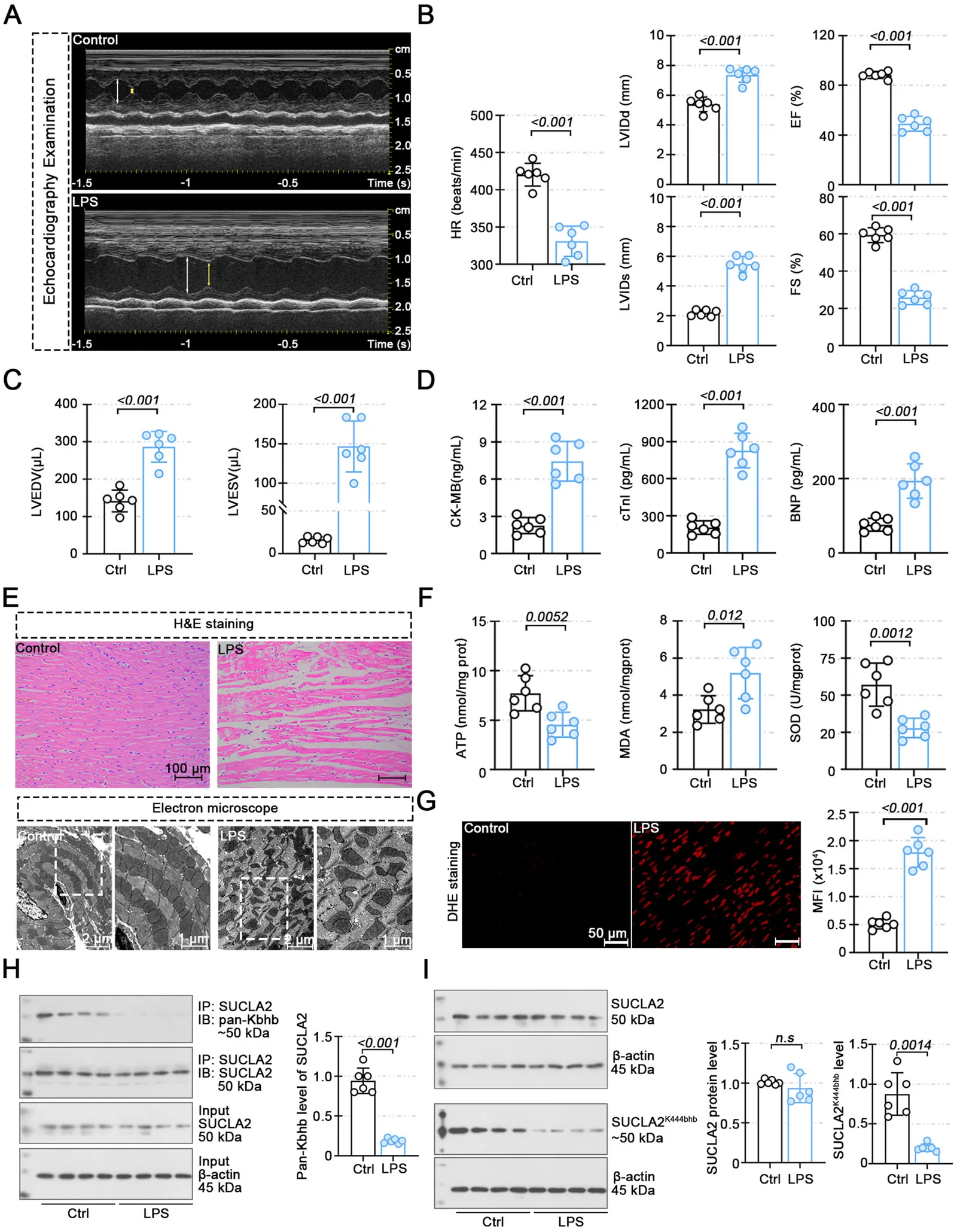
**Intraperitoneal injection of lipopolysaccharide induced sepsis-induced heart dysfunction in rats** (A) Representative echocardiographic images at 12 h post-surgery. The white line indicates the left ventricular internal dimension in diastole (LVIDd) and yellow line indicates the left ventricular internal dimension in systole (LVIDs). (B) Heart rate (HR), LVIDd, LVIDs, ejection fraction (EF), and fractional shortening (FS) in rats with sepsis-induced heart dysfunction. Biological replicates: N = 6. The statistic comparison methods are Unpaired *t*-test. (C) Left ventricular end-diastolic volume (LVEDV) and left ventricular end-systolic volume (LVESV) in rats with sepsis-induced heart dysfunction. Biological replicates: N = 6. The statistic comparison method of LVEDV is Unpaired *t*-test and the statistic comparison method of LVESV is Unpaired *t*-test with Welch's correction. (D) Detection of cardiac troponin I (cTnI), creatine kinase isoenzyme (CK-MB), and brain natriuretic peptide (BNP) levels in serum from rats with sepsis-induced heart dysfunction. Biological replicates: N = 6. The statistic comparison methods are Unpaired *t*-test. (E) Representative H&E staining and mitochondrial transmission electron microscopy images of myocardial tissues from rats with sepsis-induced heart dysfunction. (F) The ATP levels, malondialdehyde (MDA) content, and superoxide dismutase (SOD) activity in myocardial tissues from rats with sepsis-induced heart dysfunction. Biological replicates: N = 6. The statistic comparison methods are Unpaired *t*-test. (G) Representative DHE staining images of myocardial tissues from rats with sepsis-induced heart dysfunction, and corresponding mean fluorescence intensity (MFI) values. Biological replicates: N = 6. The statistic comparison method is Unpaired *t*-test with Welch's correction. (H-I) Immunoblot validation of SUCLA2 protein level, pan-Kbhb level, and specific Kbhb level at K444 site in myocardial tissues from rats with sepsis-induced heart dysfunction. Biological replicates: N = 6, The statistic comparison methods in H-I are Unpaired *t*-test with Welch's correction. Data are presented as the mean ± SD. IB, immunoblot; IP, immunoprecipitation; SUCLA2^K444bhb^, Kbhb of SUCLA2 at K444.

### 3.3 Decrease SUCLA2 Kbhb at K444 promotes cardiomyocyte mitochondrial dysfunction

The potential roles of SUCLA2 Kbhb at the K444 site were investigated *in vitro* using the H9c2 cardiomyocyte cells treated with lipopolysaccharide (Fig. 4A). Treatment of H9c2 cardiomyocytes with 10 μg/mL lipopolysaccharide for 12 h resulted in decreased SOD activity, increased MDA levels (Fig. 4B), and decreased ATP levels (Fig. 4C). The dissipation of the mitochondrial membrane potential (Fig. 4D) and increased mitochondrial ROS production levels (Fig. 4E) were also observed in lipopolysaccharide-treated H9c2 cells. A previous study demonstrated that SUCLA2 directly determines the substrate specificity of succinyl-CoA synthetase (SCS) for adenosine diphosphate/ATP through encoding its β-subunit [33]. After treatment with lipopolysaccharide, the SCS activity in H9c2 cells was significantly suppressed (Fig. 4F). In lipopolysaccharide-treated H9c2 cardiomyocytes, the pan-Kbhb levels and the Kbhb levels at the K444 site of SUCLA2 were significantly decreased with indistinctive protein change (Fig. 4G–I).

**Fig. 4:**
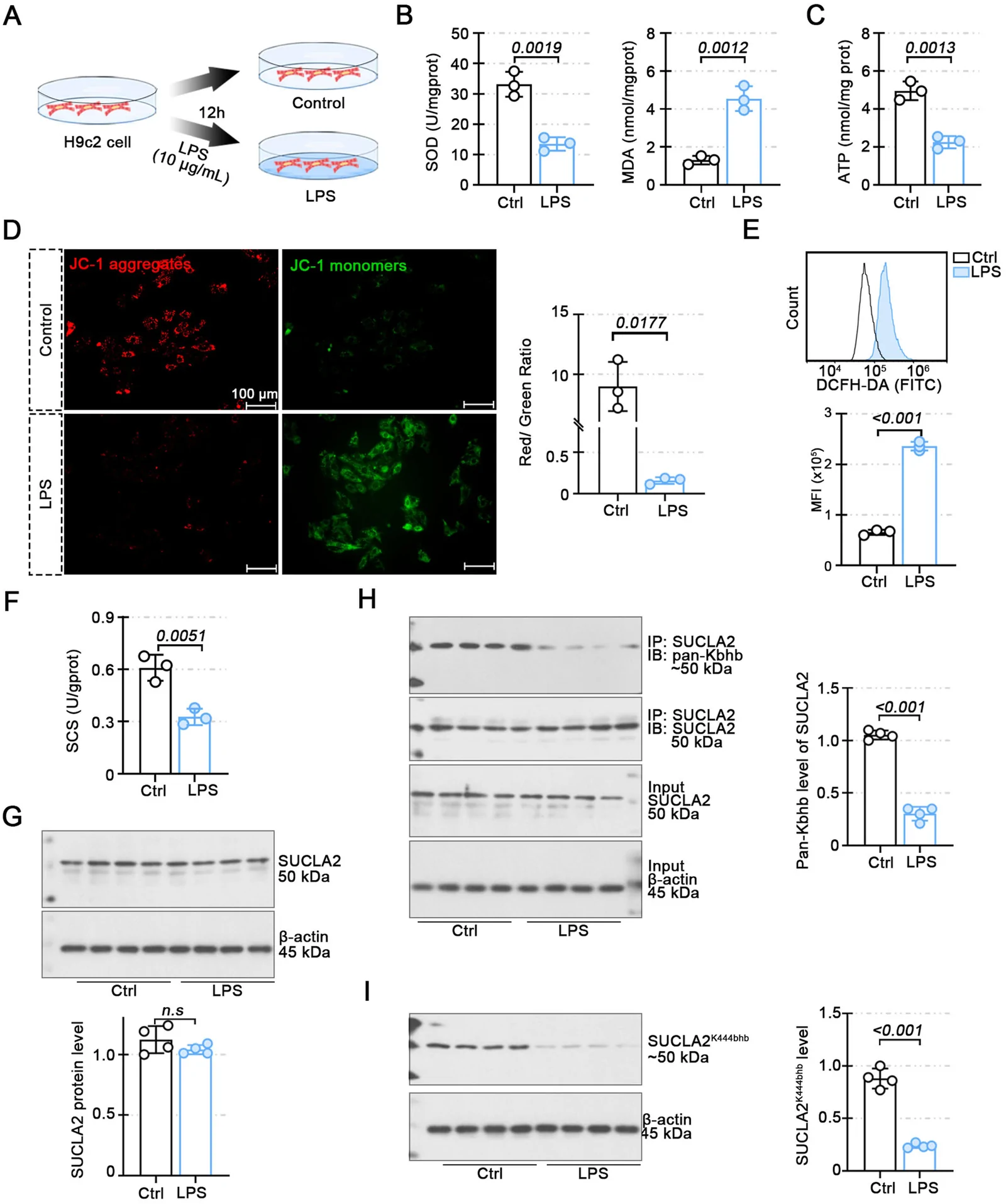
**Decreased Kbhb level of SUCLA2 at K444 site in lipopolysaccharide-treated H9c2 cardiomyocytes** (A) Schematic diagram of the experimental treatment of H9c2 cardiomyocytes. H9c2 cardiomyocytes are divided into two groups: one group is the control group without any treatment, and the other group is treated with 10 μg/mL lipopolysaccharide. (B) Determination of MDA content and SOD activity in H9c2 cardiomyocytes. Technical replicates: n = 3. The statistic comparison methods are Unpaired *t*-test. (C) ATP production in lipopolysaccharide-treated H9c2 cardiomyocytes. Technical replicates: n = 3. The statistic comparison method is Unpaired *t*-test. (D) The membrane potential of H9c2 cell mitochondria was assessed by means of the JC-1 assay. JC‐1 aggregates exhibit red fluorescence, and JC‐1 monomers exhibit green fluorescence (left), and the ratio of the mean fluorescence intensity of JC‐1 red to that of JC‐1 green fluorescence (right). Technical replicates: n = 3. The statistic comparison method is Unpaired *t*-test with Welch's correction. (E) Flow cytometry analysis of reactive oxygen species (ROS) generation in H9c2 cardiomyocytes (above), and corresponding mean fluorescence intensity (MFI) values (below). Technical replicates: n = 3. The statistic comparison method is Unpaired *t*-test. (F) Determination of SCS activity in H9c2 cardiomyocytes upon lipopolysaccharide treatment. Technical replicates: n = 3. The statistic comparison method is Unpaired *t*-test. (G) Determination of SUCLA2 protein levels in H9c2 cell lysates using Western blot assay. Technical replicates: n = 4. The statistic comparison method is Unpaired *t*-test. (H) Determination of pan-Kbhb levels of SUCLA2 in H9c2 cell lysates using coimmunoprecipitation assay. Technical replicates: n = 4. The statistic comparison method is Unpaired *t*-test. (I) Determination of Kbhb level of SUCLA2 at K444 site in H9c2 cell lysates using Western blot assay. Technical replicates: n = 4. The statistic comparison method is Unpaired *t*-test with Welch's correction. Data are presented as the mean ± SD. IB, immunoblot; IP, immunoprecipitation; SUCLA2^K444bhb^, Kbhb level of SUCLA2 at K444; n.s, no significant.

The lysine to arginine mutation mimicked reduced Kbhb levels *in vitro* [34,35]. The reduced Kbhb mimicking mutations of SUCLA2 were generated by site-directed mutagenesis of lysine 444 to arginine (Fig. 5A), which reduced the SUCLA2 Kbhb levels (Fig. 5B). We further demonstrated that the transfection of SUCLA2^K444R^ plasmids did not affect the pan-acetyllysine or pan-succinyllysine levels of SUCLA2 in lipopolysaccharide-treated H9c2 cells (Fig. S3A). SUCLA2^K444R^ plasmid transfection induced decreased mitochondrial membrane potential (Fig. 5C) and increased ROS levels (Fig. 5D and Fig. S3B) in lipopolysaccharide-treated H9c2 cells. SUCLA2^K444R^ mutant showeddecreased SCS activity and ATP production in H9c2 cardiomyocytes upon lipopolysaccharide (Fig. 5E and F). Results from the SUCLA2^K444R^ mutant cell lysate revealed a notable reduction in complex III and citrate synthase activity (Fig. 5G and H). Quantitative PCR showed that mitochondrial DNA levels had decreased by nearly half in SUCLA2^K444R^-transfected H9c2 cardiomyocytes upon lipopolysaccharide (Fig. 5I). The nuclear factor erythroid 2-related factor (NRF2) is a major regulator of the cellular defense system against oxidative stress. NRF2 binds to antioxidant response elements and initiates the transcription of the cytoprotective enzymes heme oxygenase-1 (HO-1) and NAD(P)H quinone oxidoreductase 1 (NQO1) under oxidative stress conditions [[36], [37], [38]]. Fig. 5J showed that the levels of NRF2, HO-1, and NQO1 proteins decreased significantly upon SUCLA2^K444R^ transfection in lipopolysaccharide-treated cells. These results suggest that decreased Kbhb level of SUCLA2 K444 leads to mitochondrial dysfunction and increased oxidative stress in lipopolysaccharide-treated H9c2 cells.

**Fig. 5:**
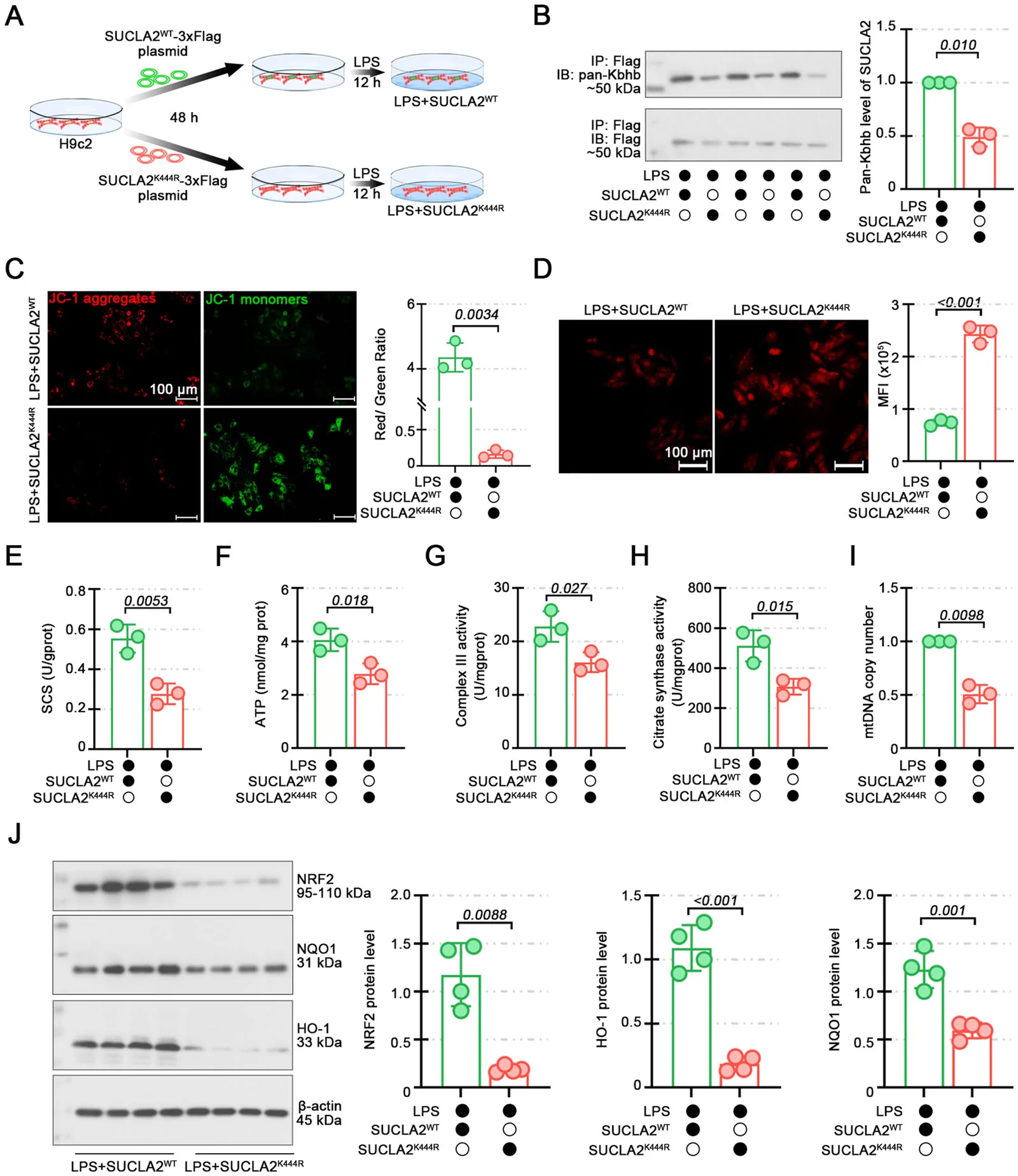
**Decreased Kbhb level of SUCLA2 K444 site mediated mitochondrial dysfunction in lipopolysaccharide-treated H9c2 cardiomyocytes** (A) Schematic diagram of the experimental treatment of H9c2 cardiomyocytes. H9c2 cardiomyocytes were transfected with wild-type (SUCLA2^WT^) or arginine mutation at K444 of SUCLA2 (SUCLA2^K444R^) plasmid for 48 h. The cells were then treated with 10 μg/mL lipopolysaccharide for 12 h. (B) Detection of SUCLA2-specific Kbhb modification levels in H9c2 cell lysates using coimmunoprecipitation assay. Technical replicates: n = 3. The statistic comparison method is Unpaired *t*-test with Welch's correction. (C) Membrane potential of H9c2 cell mitochondria was assessed by means of the JC-1 assay. JC‐1 aggregates exhibit red fluorescence, and JC‐1 monomers exhibit green fluorescence and the ratio of the MFI of JC-1 red fluorescence to that of JC-1 green fluorescence. Technical replicates: n = 3. The statistic comparison method is Unpaired *t*-test with Welch's correction. (D) Representative DHE staining images of lipopolysaccharide-treated H9c2 cardiomyocytes. Qualifications of MFI values of DHE staining images. Technical replicates: n = 3. The statistic comparison method is Unpaired *t*-test. (E) SCS activity in H9c2 cardiomyocytes. Technical replicates: n = 3. The statistic comparison method is Unpaired *t*-test. (F) Determination of ATP production in H9c2 cardiomyocytes with indicated treatments. Technical replicates: n = 3. The statistic comparison method is Unpaired *t*-test. (G) Mitochondrial Complex III activity in H9c2 cardiomyocytes with indicated treatments. Technical replicates: n = 3. The statistic comparison method is Unpaired *t*-test. (H) Citrate synthase activity in H9c2 cardiomyocytes with indicated treatments. Technical replicates: n = 3. The statistic comparison method is Unpaired *t*-test. (I) The mtDNA copy number of H9c2 cardiomyocytes with indicated treatments were determined by qPCR analysis. Technical replicates: n = 3. The statistic comparison method is Unpaired *t*-test with Welch's correction. (J) Determination of NRF2, NQO1, and HO-1 protein expression in H9c2 cell lysates using Western blot assay. Technical replicates: n = 4. The statistic comparison method of NRF2 is Unpaired *t*-test with Welch's correction and the statistic comparison methods of NQO1 and HO-1 are Unpaired *t*-test. Data are presented as the mean ± SD. IB, immunoblot; SUCLA2^WT^, wild-type SUCLA2; SUCLA2^K444R^, arginine mutation at K444 site of SUCLA2.

### 3.4 HDAC1 mediates decreased Kbhb modification of SUCLA2 at K444 site

We further investigated the potential molecular mechanisms of SUCLA2 Kbhb using IP-LC/MS (Fig. 6A). The results suggested that the histone deacetylase 1 (HDAC1) and sirtuin 2, among the identified Kbhb deacylases, could specifically interact with the SUCLA2 protein (Fig. 6B). HDAC1 promotes mitochondrial damage and increases cardiac injury caused by sepsis. Moreover, HDAC1 was associated with oxidative stress and lysine modification in lipopolysaccharide-stimulated cardiomyocytes (Fig. 6C). Double immunofluorescence staining showed the colocalization of SUCLA2 and HDAC1 in the cytoplasm of cells with or without lipopolysaccharide treatment (Fig. 6D and E). Co-immunoprecipitation and glutathione S-transferase pull-down assays revealed the protein interaction between SUCLA2 and HDAC1 (Fig. 6F and G). Molecular docking also showed high interaction ability of HDAC1 and SUCLA2 proteins (Fig. 6H). The plasmid system was constructed to induce HDAC1 knockdown in lipopolysaccharide-stimulated cardiomyocytes. HDAC1 knockdown increased Kbhb level at K444 of SUCLA2 in lipopolysaccharide-treated H9c2 cells (Fig. 6I). The results indicated that HDAC1 decreases Kbhb modification of SUCLA2 K444 site.

**Fig. 6:**
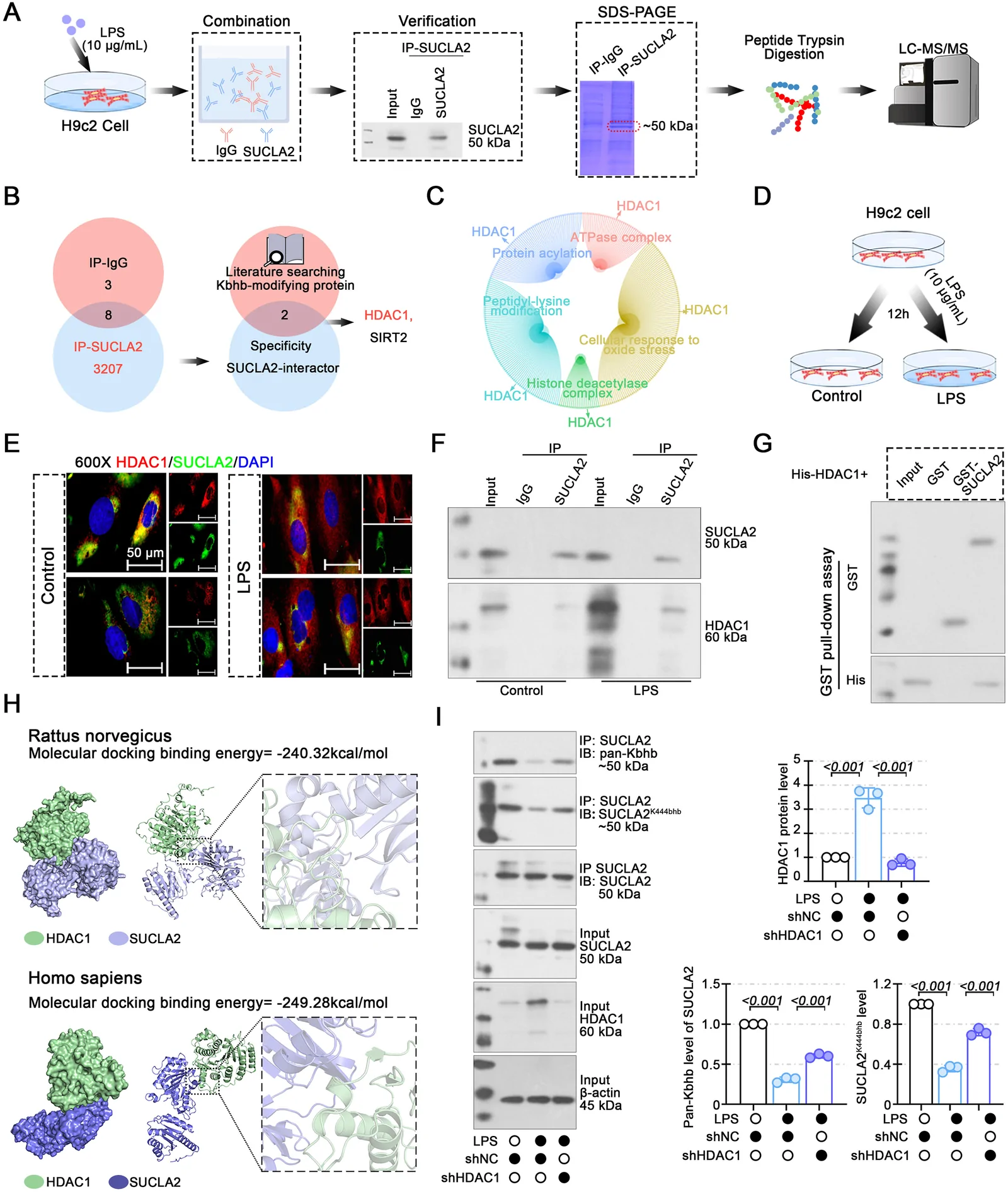
**HDAC1 decreased Kbhb level of SUCLA2 at K444 site** (A) Schematic diagram of identifying SUCLA2 interacting proteins in lipopolysaccharide-stimulated H9c2 cardiomyocytes using IP-LC/MS. (B) Venn diagram of specific SUCLA2 interaction protein and the Kbhb-modifying protein screened with literature searching. (C) Chordal graph of GO enrichment analysis of the HDAC1-related pathway of SUCLA2-specific interacting proteins. (D) Schematic diagram of the experimental treatment of H9c2 cardiomyocytes. H9c2 cardiomyocytes were lipopolysaccharide at a concentration of 10 μg/mL for 12 h. (E) Confocal microscopy demonstrating the colocalization of HDAC1 with SUCLA2. (F) Co-immunoprecipitation analysis of the interaction of SUCLA2 with HDAC1 in H9c2 cardiomyocytes. (G) Glutathione S-transferase pull-down assay was performed to determine the interaction between HDAC1 and SUCLA2 *in vitro*. (H) Molecular docking between SUCLA2 and HDAC1 in *Rattus norvegicus* and *Homo sapiens*. (I) Coimmunoprecipitation analysis of the Kbhb modification levels of SUCLA2 in HDAC1 knockdown H9c2 cardiomyocytes. Technical replicates: n = 3. The statistic comparison methods are One-way ANOVA with Tukey test. Data are presented as the mean ± SD. IB, immunoblot; SUCLA2^WT^, wild-type SUCLA2; SUCLA2^K444R^, arginine mutation at K444 site of SUCLA2; SUCLA2^K444bhb^, Kbhb level of SUCLA2 at K444.

### 3.5 Decreased SUCLA2 Kbhb level at K444 aggravates mitochondrial dysfunction of cardiomyocytes *in vivo*

In order to verify the experimental results conducted *in vitro*, the AAV9 vectors containing a cardiomyocyte-specific promoter was used to express SUCLA2^WT^ and SUCLA2^K444R^ *in vivo* (Fig. S4A). There were no marked differences among the Vector, SUCLA2^WT^, and SUCLA2^K444R^ groups, likely because cardiac function was unchanged without lipopolysaccharide treatment (Fig. S4B–S4D). The septic heart dysfunction rat model was further established after 2 weeks of AAV9 infection (Fig. 7, Fig. 8A). Flag expression confirmed that AAV9 markedly induced SUCLA2^WT^ and SUCLA2^K444R^ expressions in the cardiomyocytes of myocardial tissues of rats treated with lipopolysaccharide (Fig. 7B). SUCLA2^WT^ transfection alleviated heart dysfunction in rats, while SUCLA2^K444R^ group showed poorer heart function and cardiac hemodynamics (Fig. 7C–E). The decreased serum levels of CK-MB, cTnI, and BNP, triggered by SUCLA2^WT^ transfection, were reversed upon SUCLA2^K444R^ transfection (Fig. 7F). Pathological histological examination and mitochondrial function were assessed from isolated myocardial tissue from rats of different groups. SUCLA2^K444R^ group exhibited disorganized myocardial fiber arrangement and severe tissue damage *in vivo* (Fig. 8B). Mitochondrial damage mitigated by SUCLA2^WT^ was exacerbated following SUCLA2^K444R^ transfection in the myocardial tissue of septic rats (Fig. 8B). The ROS in rat myocardial tissues was significantly accumulated following SUCLA2^K444R^ transfection (Fig. 8B and C). The SUCLA2^WT^ group showed increased ATP levels in the myocardial tissue of rats with sepsis, while the SUCLA2^K444R^ group exhibited lower ATP levels (Fig. 8D). The increased SOD activity and decreased MDA levels in myocardial tissue of sepsis rats, induced by SUCLA2^WT^ injection, were reversed in the SUCLA2^K444R^ group (Fig. 8E). These findings indicate that reduced Kbhb levels of SUCLA2 K444 site exacerbate sepsis-induced heart dysfunction *in vivo*.

**Fig. 7:**
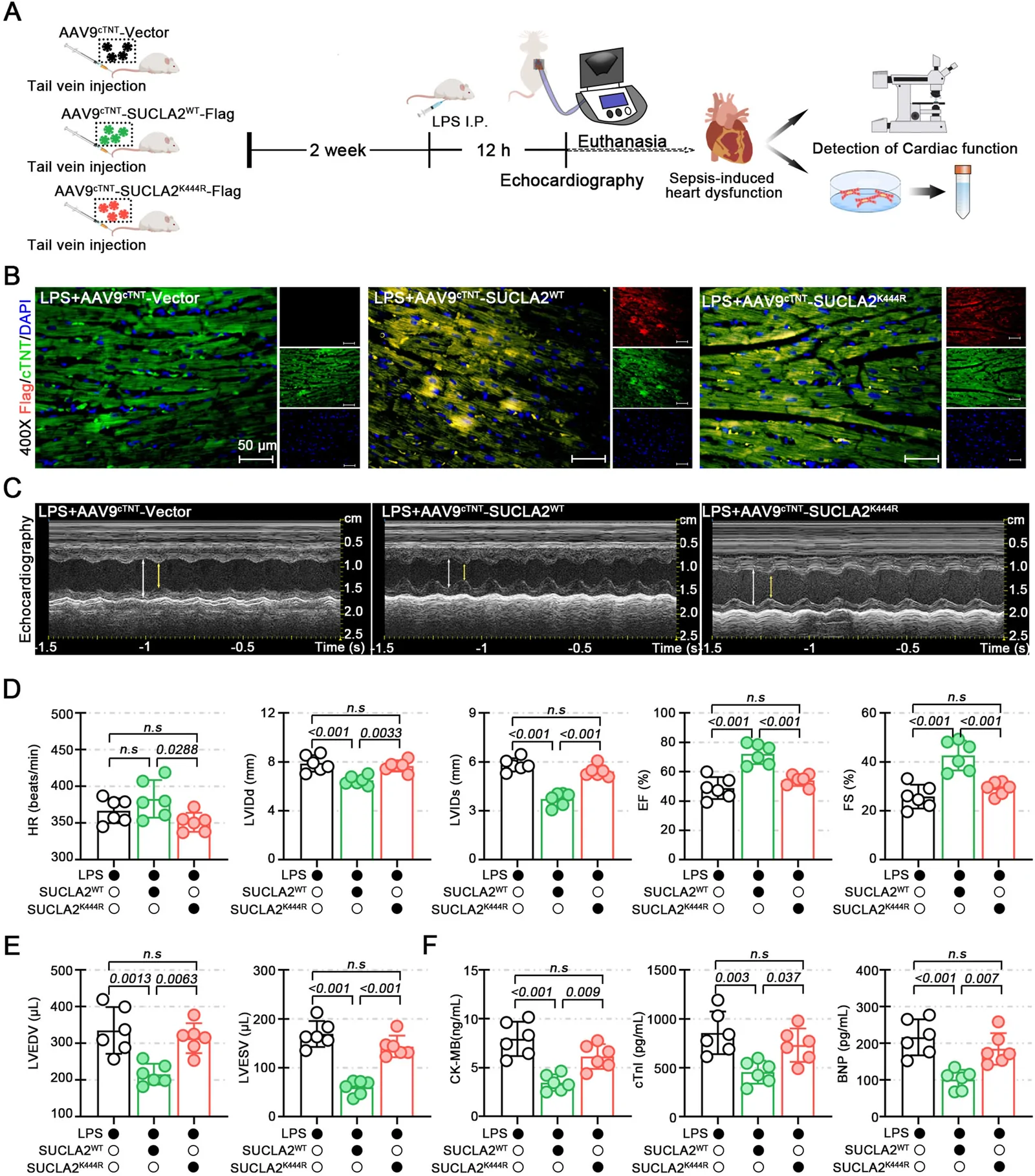
**Reduced Kbhb modification of SUCLA2 at K444 site aggravates sepsis-induced heart dysfunction** (A) Schematic diagram of the experimental procedure. Rats were tail vein injected with wild-type or K444R mutation SUCLA2 adeno-associated virus. After 2 weeks, rats were intraperitoneally injected with lipopolysaccharide. After 12 h, echocardiography was performed. The cardiac tissues were collected for molecular biological detection. (B) SUCLA2 (Flag, red) protein is localized with myocardial cell (cTNT, green) in rat myocardial tissues. (C) Representative echocardiographic images. White line indicates the LVIDd and yellow line indicates the LVIDs. (D) HR, LVIDd, LVIDs, EF, and FS in rats with sepsis-induced heart dysfunction. Biological replicates: N = 6, The statistic comparison methods are One-way ANOVA with Tukey test. (E) LVEDV and LVESV in rats with sepsis-induced heart dysfunction. Biological replicates: N = 6, The statistic comparison methods are One-way ANOVA with Tukey test. (F) Detection of cTnI, CK-MB, and BNP levels in serum from rats with sepsis-induced heart dysfunction. Biological replicates: N = 6. The statistic comparison methods are One-way ANOVA with Tukey test. Data are presented as the mean ± SD. IB, immunoblot; SUCLA2^WT^, wild-type SUCLA2; SUCLA2^K444R^, arginine mutation at K444 site of SUCLA2; n.s, no significant.

**Fig. 8:**
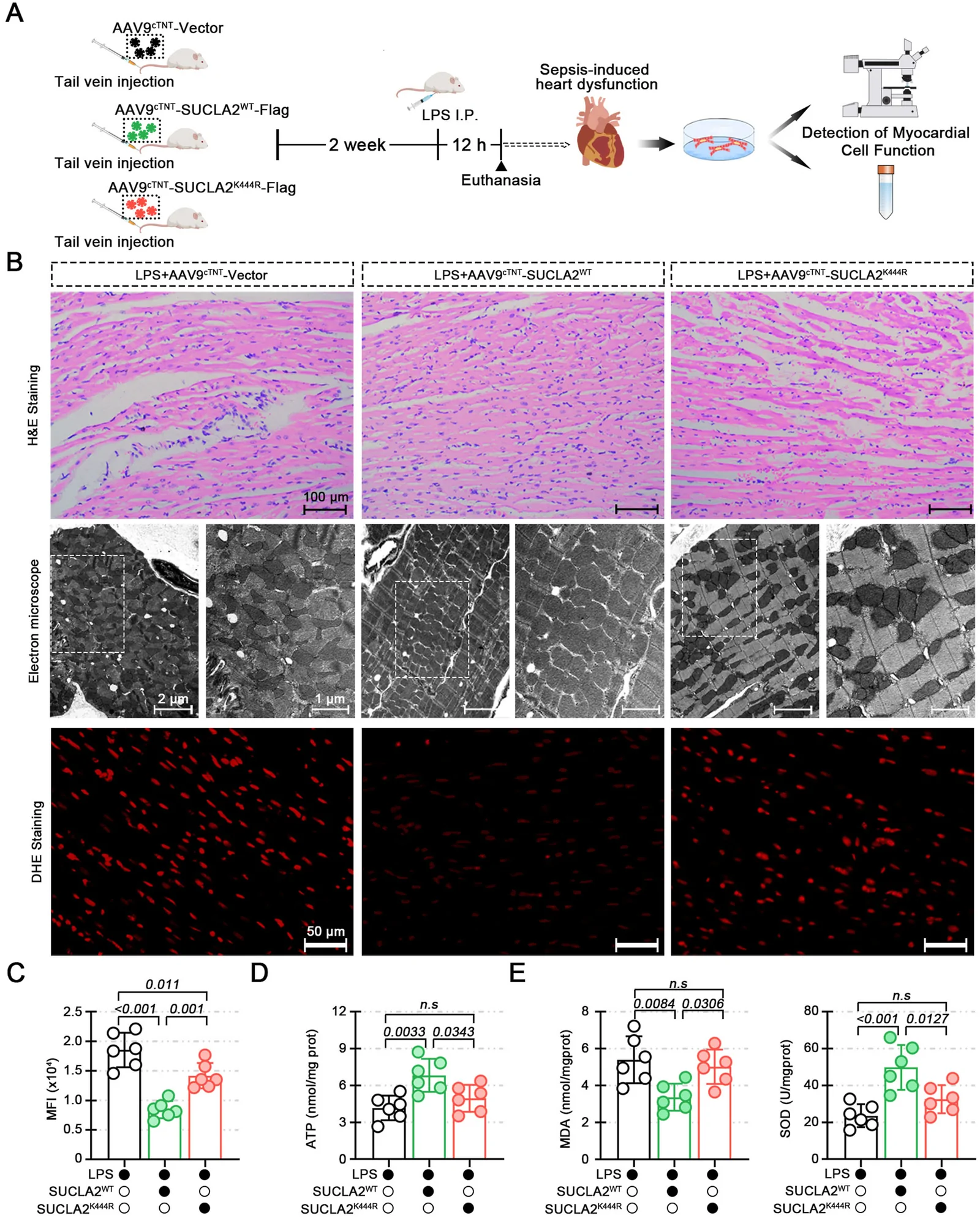
**Reduced Kbhb modification of SUCLA2 at K444 site aggravates histopathological changes in sepsis-induced cardiomyopathy *in vivo*** (A) Schematic diagram of the experimental procedure. Rats were tail vein injected with wild-type or K444R mutant SUCLA2 virus. After 2 weeks, rats were then intraperitoneally injected with lipopolysaccharide to induce sepsis. After 12 h, echocardiography is performed. The cardiac tissues are collected for pathological examination. (B) Representative H&E staining, mitochondrial transmission electron microscopy, and DHE staining images of myocardial tissues from rats with sepsis-induced heart dysfunction. (C) Corresponding MFI values of DHE staining images in (B). Biological replicates: N = 6. The statistic comparison method is One-way ANOVA with Tukey test. (D) The ATP production level in myocardial tissues from rats. Biological replicates: N = 6, The statistic comparison method is One-way ANOVA with Tukey test (E) The MDA content and SOD activity in myocardial tissues from rats. Biological replicates: N = 6, The statistic comparison method is One-way ANOVA with Tukey test. Data are presented as the mean ± SD. SUCLA2^WT^, wild-type SUCLA2; SUCLA2^K444R^, arginine mutation at K444 residue of SUCLA2.

### 3.6 Transcriptomic analysis predicts potential mechanism of SUCLA2 Kbhb *in vitro*

The potential mechanism of Kbhb at K444 of SUCLA2 was investigated using the transcriptomic analysis. As shown in Fig. 9A, 48 h after plasmid transfection, the H9c2 cells were treated with lipopolysaccharide for 12 h. Then, RNA was extracted and the library was constructed for sequencing. Principal component analysis and heatmap analysis revealed distinct clustering patterns between different groups (Fig. 9B). A total of 1829 differentially expressed mRNAs were identified (Fig. 9C), and heatmaps were used to display the overlapping differentially expressed mRNAs between the groups (Fig. 9D). KEGG enrichment analysis showed that the IL-17 signaling pathway, MAPK signaling pathway, TNF signaling pathway, and AMPK signaling pathway were significantly enriched (Fig. 9E). Several biological processes enriched by GO analysis were linked to sepsis-induced heart dysfunction (Fig. 9F), including cellular response to reactive oxygen species, cellular response to oxygen levels, cellular response to tumor necrosis factor, and regulation of DNA-templated transcription in response to stress (Fig. 9G). Deng et al. demonstrated that the deficiency release of IL-17A in bone marrow-derived dendritic cells inhibits the nitric oxide through the JNK/AP1 pathway, thereby protecting cardiomyocytes from death in sepsis-induced myocardial dysfunction [39]. l-carnitine alleviates oxidative stress, improves mitochondrial damage, and ameliorates lipopolysaccharide-induced injury to cardiomyocytes via the JUN/MAPK signaling pathway [40]. Sepsis triggers the activation of JUN, which induces the production of the pro-inflammatory cytokine TNFα, aggravating the cardiac dysfunction caused by sepsis [41]. Possible mechanisms by which Kbhb of SUCLA2 K444 contributes to sepsis-induced cardiac dysfunction at the were investigated, and further experiments are needed to validate.

**Fig. 9:**
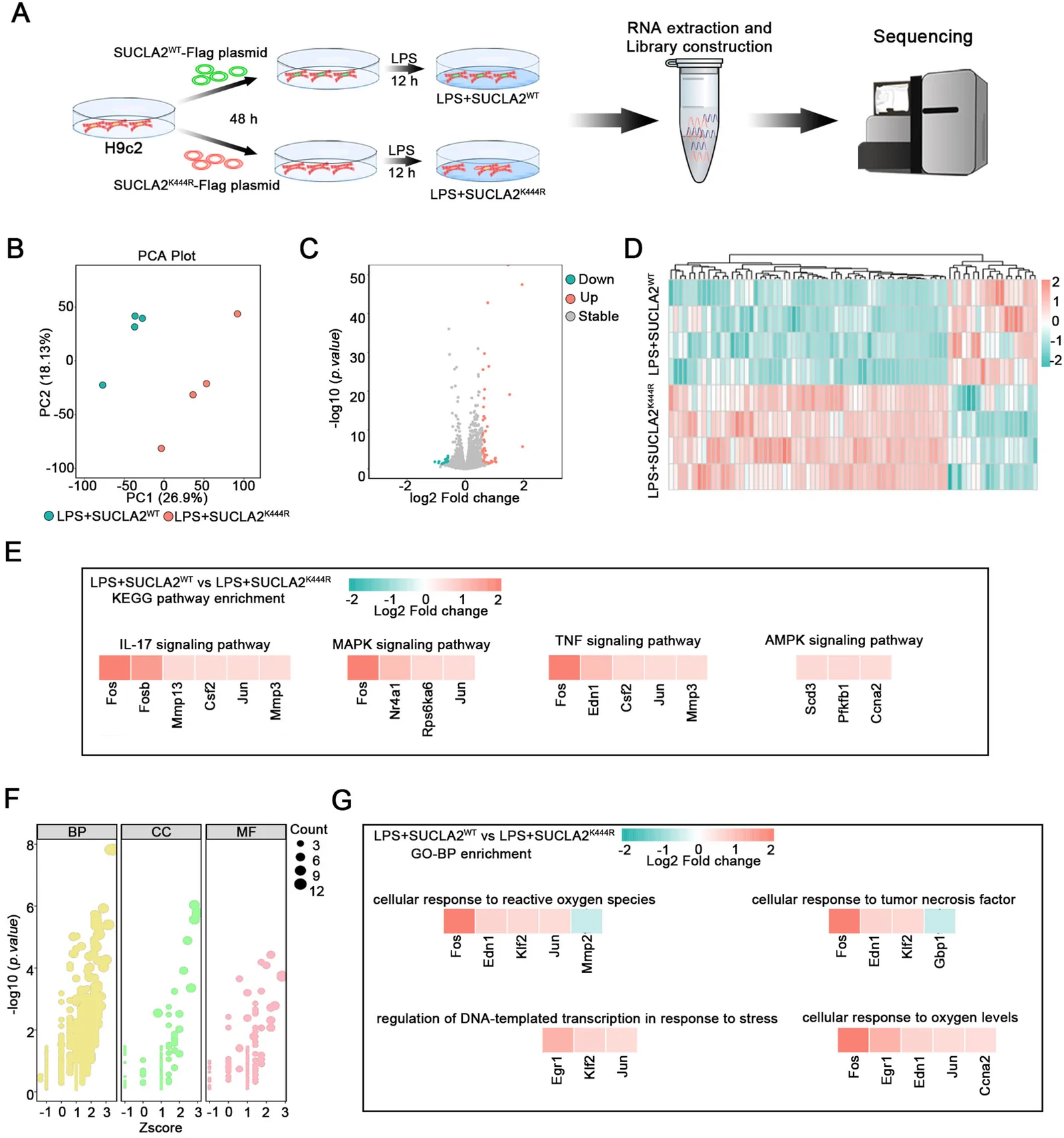
**Transcriptomic analysis speculates potential mechanism of SUCLA2 Kbhb *in vitro*** (A) Schematic diagram of the transcriptomic analysis procedure. (B) Principal component analysis showing the differences between the wild-type and K444R mutation model group group. (C) Volcano plot depicting the upregulated and downregulated genes in lipopolysaccharide-stimulated H9c2 cardiomyocytes. Technical replicates: n = 4. (D) Heatmap depicting the upregulated and downregulated genes in lipopolysaccharide-stimulated H9c2 cardiomyocytes. (E) Gene lists of enrich genes with the fold change in sepsis-induced cardiomyopathy-related KEGG categories. Technical replicates: n = 4. (F) GO annotation analysis of DEGs in H9c2 cardiomyocytes under various treatments. (G) Gene lists of enrich genes with the fold change in sepsis-induced cardiomyopathy-related GO categories.

## 4 Discussion

The molecular and biological processes related to septic heart dysfunction include oxidative stress, insufficient ATP, alterations in metabolism, and mitochondrial dysfunction [42,43]. Kbhb has been verified to regulate mitochondrial function as a post-translational modification in various diseases [18,44]. Nevertheless, the correlation between Kbhb modification and sepsis-induced heart dysfunction necessitates additional investigation.

In this study, the results of proteomic and β-hydroxybutyrylomic profiling indicated the potential role of Kbhb in sepsis-induced heart dysfunction. The novel hybrid PLM-based framework was developed to predict the functional Kbhb sites. The model exhibited superior performance compared with models based on individual features. The results indicated that the Kbhb of SUCLA2 might function in sepsis-induced heart dysfunction, particularly at the K444 site. It has been reported that SUCLA2 deficiency contributes to the progression of various mitochondrial-related diseases [25]. SUCLA2 regulates the expression of antioxidant enzymes, ameliorating the oxidative stress response [45]. In the rat model of sepsis-induced heart dysfunction, we confirmed that the reduced Kbhb levels of SUCLA2 K444 site in myocardial tissues without protein expression changed. UVSLT2 is a Kbhb protein, and its Kbhb site mutation reduces fungal virulence by decreasing enzyme activity without affecting protein expression [46]. Therefore, we hypothesize that decreased Kbhb levels at K444 of SUCLA2 contribute to the progression of septic cardiomyopathy by affecting its enzymatic activity.

Previous study verified that the upregulation of SUCLA2 maintained mitochondrial function and plays a protective role in heart diseases [47]. The same results were observed in this study: SUCLA2 alleviated lipopolysaccharide-induced mitochondrial dysfunction in H9c2 cells. The point K to R mutant of SUCLA2 K444 site was constructed to mimic reduced Kbhb levels, according to previous studies [13,46,48]. The results showed that the SUCLA2^K444R^ mutant reversed the protective effect of SUCLA2 on damaged cardiac cells by enhancing mitochondrial dysfunction. The *in vitro* results suggested that reduced Kbhb at K444 site of SUCLA2 aggravates mitochondrial dysfunction in injured cardiac cells. We further verified the role of SUCLA2 Kbhb at K444 *in vivo*. The AAV9 vector containing the cTnT promoter was used to express SUCLA2^WT^ and SUCLA2^K444R^ in the cardiomyocytes of septic rats. Myocardial cell membrane damage releases CK-MB and cTnI, classical protein markers of myocardial necrosis, into the bloodstream [49]. Excessive volume load or heart failure causes ventricular muscle cells to secrete large amounts of BNP [50]. The SUCLA2^WT^ transfection decreased serum CK-MB, cTnI, and BNP levels, which were reversed by the SUCLA2^K444R^ transfection. Combining the results with structural observations indicated that cardiac function was significantly better in the SUCLA2^WT^ group than in the vector group in the rat model of sepsis-induced heart dysfunction. The SUCLA2^K444R^ group experienced impaired cardiac function accompanied by exacerbated mitochondrial dysfunction. These results suggest that decreased Kbhb levels of SUCLA2 K444 site aggravates sepsis-induced heart dysfunction.Surprisingly, the overexpression of SUCLA2^K444R^ in cardiomyocytes in rats did not significantly depress mitochondrial function or cause cardiac depression in the absence of lipopolysaccharide. We hypothesize that the mismatch between lipopolysaccharide-driven energy demand and SUCLA2^K444R^-impaired mitochondrial ATP production exacerbates disease progression. In the research of Kong et al., the K679R mutation does not affect macrophage inflammation without lipopolysaccharide treatment, while this mutation completely abolishes the anti-inflammatory effects under lipopolysaccharide treatment [51]. Additional experiments are required to validate the above hypothesis and refine the relevant conclusions.

We further performed IP-LC/MS analyses to elucidate the potential molecular mechanisms that modulate Kbhb of SUCLA2. HDAC1 was identified as a potential enzyme in regulating SUCLA2 Kbhb. HDAC1 has been shown to localize within the mitochondrial compartment [52], and downregulated HDAC1 expression reversed myocardial damage in septic mice [53]. The inhibition of HDAC1 was also shown to be a potential therapeutic strategy for mitigating mitochondrial damage [54]. HDAC1 was verified to enzymatically remove Kbhb [55]. Our data suggested that HDAC1 interacts with SUCLA2. The Kbhb level at the K444 site of SUCLA2 was increased after HDAC1 knockdown *in vitro*. The results suggest that HDAC1 decreased SUCLA2 Kbhb in cardiomyocytes. The transcriptomic analysis was performed to identify the potential mechanisms of SUCLA2 Kbhb under pathological conditions. The results showed that the mutation affected oxidative stress in cardiomyocytes, which is consistent with our previous findings. Furthermore, the enriched pathways: IL-17 signaling pathway [39], MAPK signaling pathway [56], TNF signaling pathway [57], and AMPK signaling pathway [58] have been reported in sepsis-induced heart dysfunction. We found that JUN, which has received extensive attention as a potential target for drugs to treat sepsis and related organ damage [59], was involved in multiple pathways. The results of the aforementioned pathway analysis suggest that the SUCLA2 K444R mutation might contribute to the progression of sepsis-induced heart dysfunction by affecting downstream genes (such as JUN), which needs further validation.

We acknowledge several limitations that contextualize our findings and will guide future research. We primarily performed our detailed mechanistic studies in rat and cell models because samples of septic and normal control human myocardial injury were difficult to obtain.

## 5 Conclusion

This study presents the landscape of protein Kbhb in sepsis-induced heart dysfunction. We confirmed that a decreased SUCLA2 Kbhb level, mediated by HDAC1, results in increased mitochondrial dysfunction and decreased heart function, both *in vitro* and *in vivo*. These results offer new insights into the pathophysiology of sepsis-induced heart dysfunction.

## CRediT authorship contribution statement

**Ri Wen:** Data curation, Formal analysis, Writing – original draft. **Wanshan Ning:** Formal analysis, Methodology, Investigation, Writing – original draft. **Xin-Mei Huang:** Data curation, Formal analysis, Writing – original draft. **Peng-Hui Hao:** Data curation, Investigation, Software, Writing – original draft. **Jingjing Yang:** Data curation, Investigation, Writing – original draft. **Wei Liu:** Data curation, Formal analysis, Writing – review & editing. **Yu-Hang Yang:** Formal analysis, Supervision, Writing – review & editing. **Chun-Feng Liu:** Formal analysis, Writing – review & editing. **Ni Yang:** Conceptualization, Funding acquisition, Supervision, Writing – review & editing. **Yaping Guo:** Formal analysis, Supervision, Visualization, Writing – review & editing. **Tie-Ning Zhang:** Conceptualization, Funding acquisition, Resources, Supervision, Writing – review & editing.

## Data availability

The source code and models of FuncKbhb are available at: https://github.com/kongxianzw/Kbhb. The dataset of β-hydroxybutyrylomic profiling is available in the iPROX repository (IPX0016076002).

## Funding

This work was supported by the 10.13039/501100001809National Natural Science Foundation of China (82502630 to Ri Wen, 32400532 to Wanshan Ning ), the National Key R & D Program of China (2022YFC2704300 and 2021ZD0201300 to Wanshan Ning), the 10.13039/501100005047Natural Science Foundation of Liaoning Province (2024-MS-077 to Tie-Ning Zhang, 2024-MS-067 to Ni Yang, and 2024-BSLH-329 to Ri Wen), the Fujian Science and Technology Program Guiding Project (2025D022 to Wanshan Ning), the 10.13039/501100017686Fujian Provincial Health Technology Project (2024GGB18 to Wanshan Ning)( , the Natural Science Foundation of the Shanghai Science and Technology Innovation Action Plan (24ZR1457900 to Xinmei Huang), and 345 Talent Program of Shengjing Hospital of China Medical University (M1308 to Tie-Ning Zhang and M1404 to Ni Yang).

## Declaration of competing interest

The authors declare that they have no known competing financial interests or personal relationships that could have appeared to influence the work reported in this paper.
