# Supplementary material for: HDAC1-mediated SUCLA2 lysine β-hydroxybutyrylation modulates myocardial mitochondrial function in sepsis-induced heart dysfunction
Source: Redox Biol. 2026 Oct 8;98:104431. doi: 10.1016/j.redox.2026.104431 (PMC13683460; doi:10.1016/j.redox.2026.104431)
Supplement: Supplementary file 1 [file mmc1.pdf]

HDAC1-mediated SUCLA2 lysine β-hydroxybutyrylation modulates myocardial mitochondrial function in sepsis-induced heart dysfunction

Ri Wen[a#], Wanshan Ning[b#], Xin-Mei Huang[c#], Peng-Hui Hao[a], Jingjing Yang[d], Wei Liu[e], Yu-Hang Yang[a], Chun-Feng Liu[a], Ni Yang[a*], Yaping Guo[e*], Tie-Ning Zhang[a*]

Author Affiliations

a Department of Paediatrics, PICU, Shengjing Hospital of China Medical University, Shenyang 110004, China.

b Institute for Clinical Medical Research, the First Affiliated Hospital of Xiamen University, School of Medicine, Xiamen University, Xiamen 361003, China.

c Department of Endocrinology, Shanghai Fifth People's Hospital, Fudan University, Shanghai 200240, China.

d Department of Pulmonary and Critical Care Medicine, the First Affiliated Hospital of Xiamen University, School of Medicine, Xiamen University, Xiamen 361003, China.

e Department of Pathophysiology, School of Basic Medical Sciences, Zhengzhou University, Zhengzhou 450001, China.

[#]The authors contributed equally.

*** Address for correspondence**

Ni Yang, Department of Paediatrics, PICU, Shengjing Hospital of China Medical University, No.36, SanHao Street, Shenyang City, Liaoning Province, 110004, China. Phone: +86-18940257621. E-mail: yangni616@hotmail.com

AND

Yaping Guo, Department of Pathophysiology, School of Basic Medical Sciences, Zhengzhou University, Science Avenue 100, Zhengzhou City, Henan Province, 450001, China. Phone: +86-13125139082. E-mail: guoyaping@zzu.edu.cn

AND

Tie-Ning Zhang, Department of Paediatrics, PICU, Shengjing Hospital of China Medical University, No.36, SanHao Street, Shenyang City, Liaoning Province, 110004, China. Phone: +86-18940253339. E-mail: cmuztn@vip.qq.com

Supplementary Figures

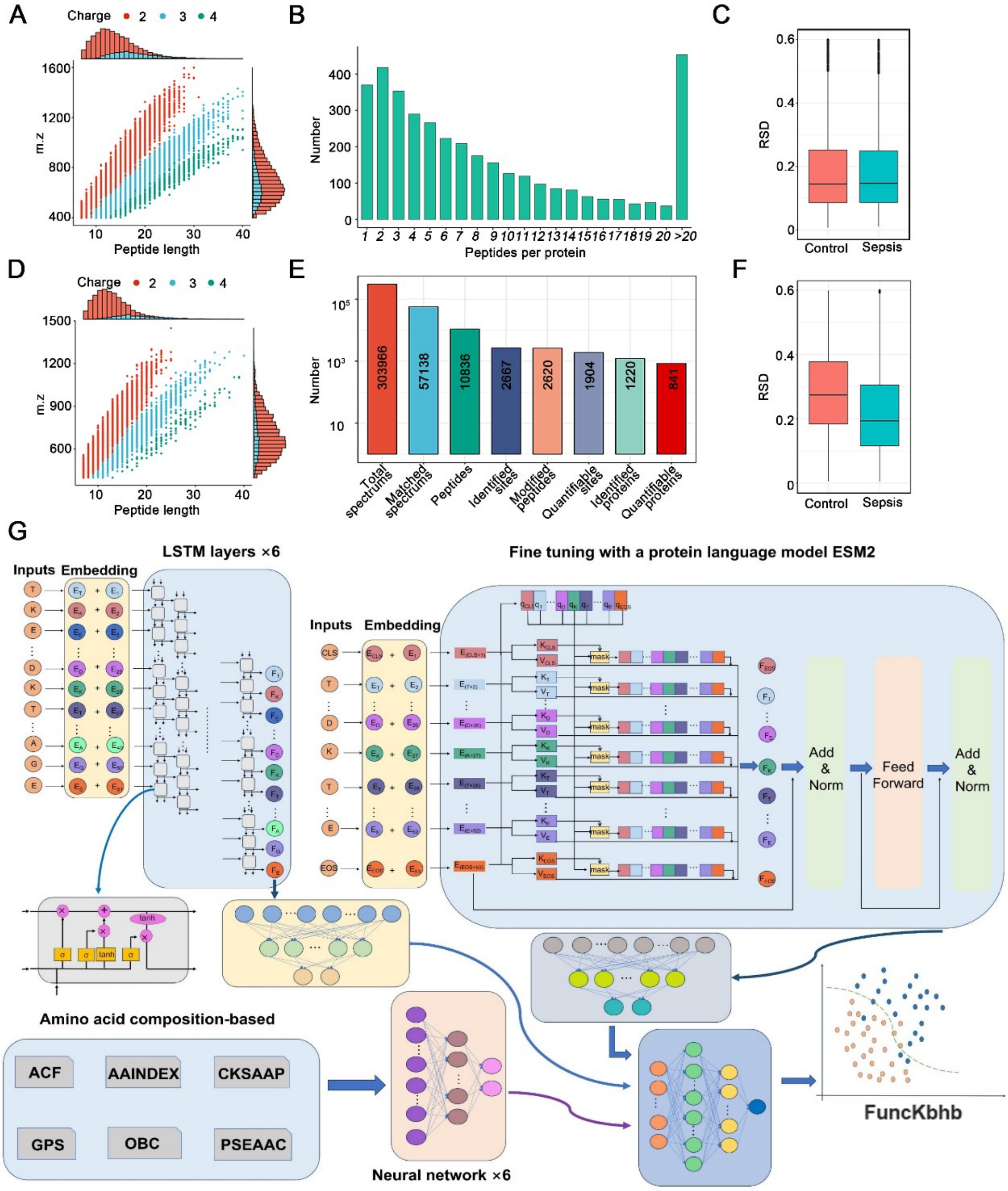

Supplemental Figure 1 Quality control and data characteristics of proteomic and β-hydroxybutyrylomic analyses and predictive modeling of Kbhb

(A, D) The length distribution of peptides identified in proteome (A) and β-hydroxybutyrylome (D). (B) The distribution of peptide numbers of quantified proteins. (C, F) Relative standard deviation distribution of proteome (C) and β-hydroxybutyrylome (F) data. (E) Summary statistics of protein and modification identification. (G) Predictive framework integrating multi-feature hybrid learning and transformer-based strategies for Kbhb site identification.

A

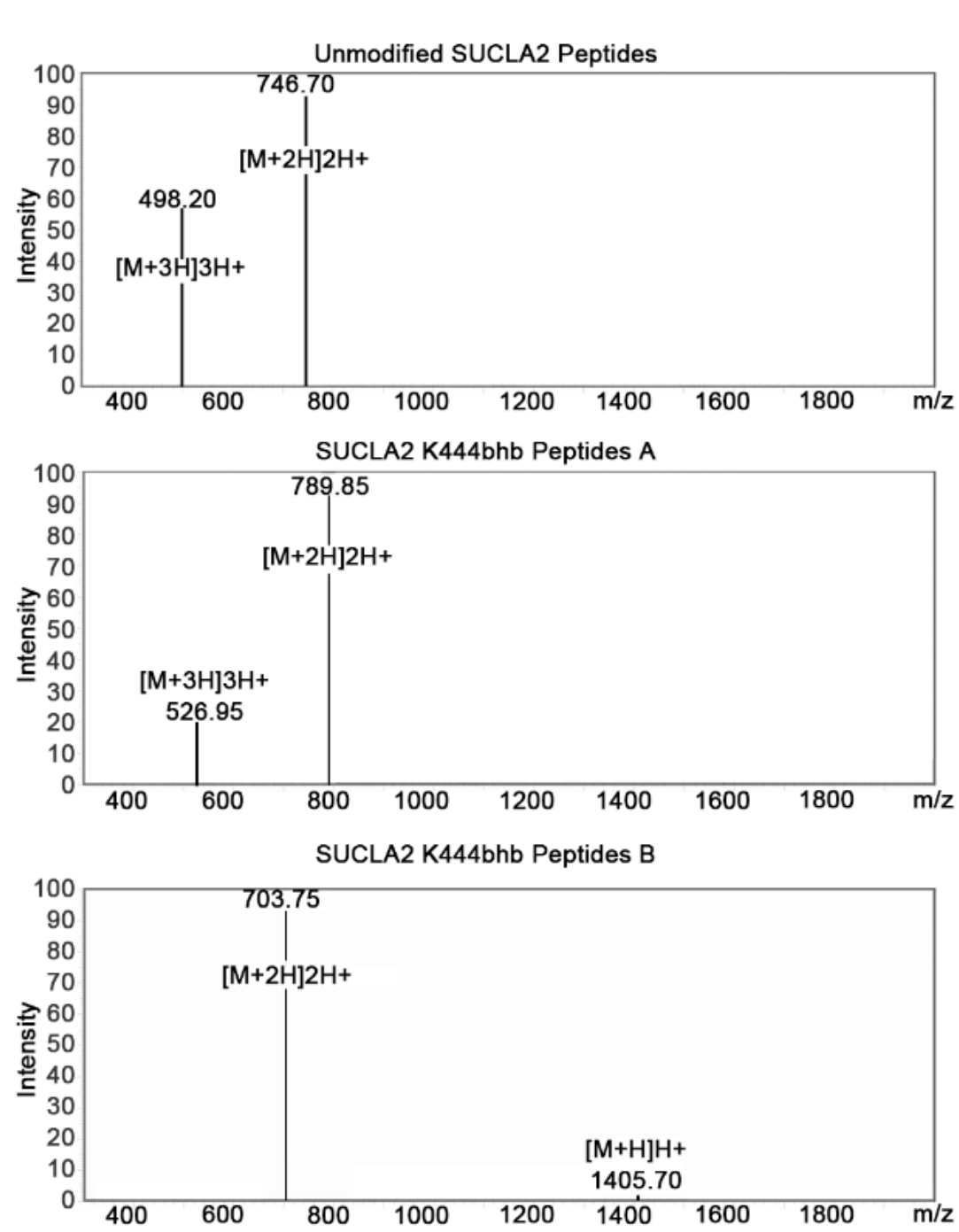

C

Dilution \ Coat \ Antibody	Ab3 Peptides A	Ab3 Peptides B	Ab3 Unmodified Peptides	Ab4 Peptides A	Ab4 Peptides B	Ab4 Unmodified Peptides
1:2K	2.45	2.57	1.725	2.509	2.596	0.21
1:6K	1.993	1.934	1.218	1.932	2.296	0.115
1:18K	1.773	1.722	0.731	1.079	1.603	0.101
1:54K	1.31	1.359	0.265	0.457	0.848	0.072
1:162K	0.707	0.658	0.107	0.148	0.361	0.079
1:486K	0.327	0.366	0.041	0.046	0.127	0.076
1:1458K	0.196	0.166	0.018	0.034	0.064	0.076
	0.062	0.012	0.018	0.013	0.025	0.075

D

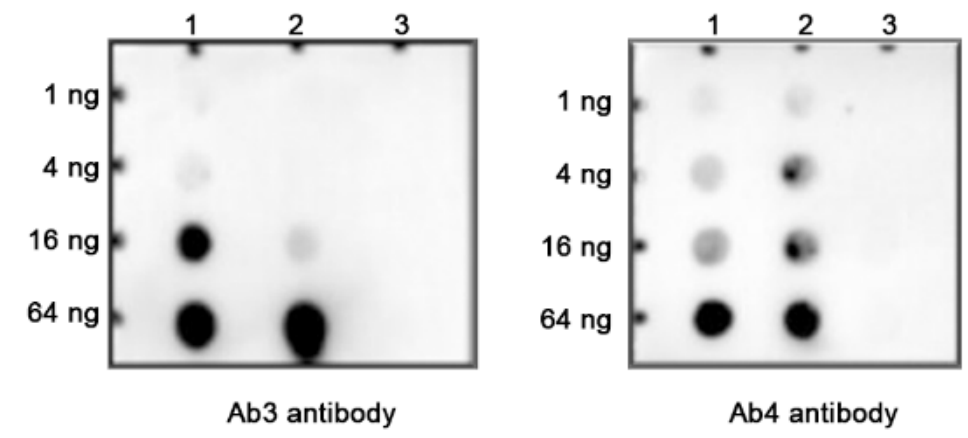

Lane 1 : SUCLA2 K444bhb Peptides A

Lane 2 : SUCLA2 K444bhb Peptides B

Lane 3 : Unmodified SUCLA2 Peptides

Primary antibody: Ab3 1:5000; Ab4 1: 2000

B

Dilution \ Rabbit \ Coat	SUCLA2 K444bhb Peptides A R1	SUCLA2 K444bhb Peptides A R2	SUCLA2 K444bhb Peptides B R1	SUCLA2 K444bhb Peptides B R2	Unmodified SUCLA2 Peptides R1	Unmodified SUCLA2 Peptides R2
1:250	1.656	1.613	0.536	0.251	1.533	1.635
1:1K	1.015	0.918	0.231	0.137	0.922	0.95
1:4K	0.444	0.349	0.117	0.097	0.411	0.341
1:16K	0.128	0.105	0.091	0.085	0.097	0.097
1:64K	0.046	0.037	0.073	0.078	0.042	0.044
1:256K	0.028	0.028	0.076	0.078	0.022	0.026
1:1024K	0.028	0.023	0.093	0.091	0.035	0.045
Blank	0.018	0.022	0.082	0.074	0.01	0.014

Dilution \ Rabbit \ Coat	SUCLA2 K444bhb Peptides A R3	SUCLA2 K444bhb Peptides A R4	SUCLA2 K444bhb Peptides B R3	SUCLA2 K444bhb Peptides B R4	Unmodified SUCLA2 Peptides R3	Unmodified SUCLA2 Peptides R4
1:250	2.531	2.476	2.615	2.524	1.658	1.153
1:1K	2.288	1.993	2.425	0.266	1.149	0.519
1:4K	1.702	1.332	1.847	1.586	0.426	0.152
1:16K	0.965	0.482	0.996	0.707	0.121	0.044
1:64K	0.31	0.138	0.352	0.238	0.04	0.013
1:256K	0.084	0.031	0.102	0.067	0.019	0.009
1:1024K	0.026	0.013	0.03	0.031	0.013	0.007
Blank	0.008	0.003	0.004	0.011	0.008	0.006

E

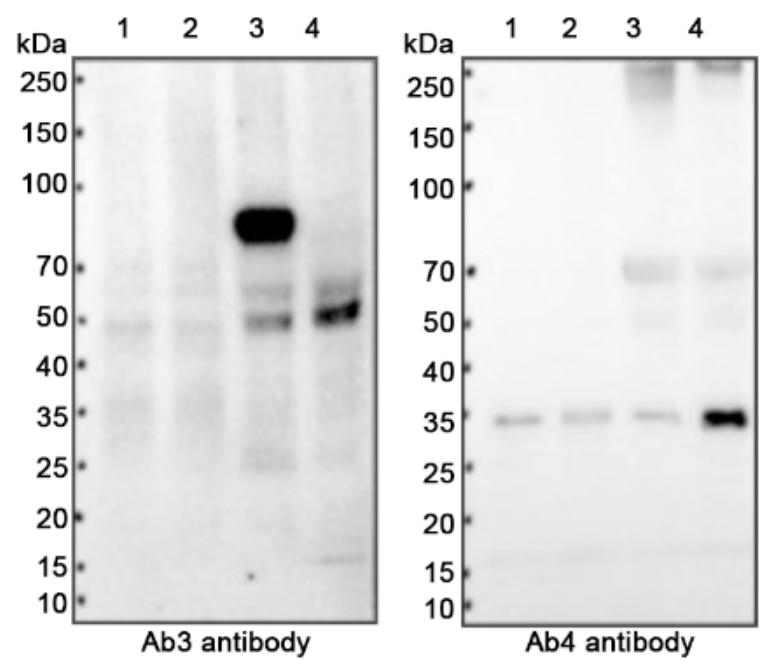

Lane 1 : C6

Lane 2 : C6+Lac Na (25mM. 24h)

Lane 3 : PC-12

Lane 4 : PC-12+Lac Na (25mM. 24h)

Primary antibody: 1: 500

Supplemental Figure 2 The validation of SUCLA2K444bhb specific antibody

(A) Mass spectrometry analysis of immunogens. (B) Preliminary ELISA screening of antisera. Rabbits immunized with Sucla2 K444bhb Peptide A were identified as R1 and R2. Rabbits immunized with Sucla2 K444bhb Peptide B were identified as R3 and R4. (C) Antibody quality control using ELISA assay. (D) Antibody dot blot detection. (E) Antibody western blot detection.

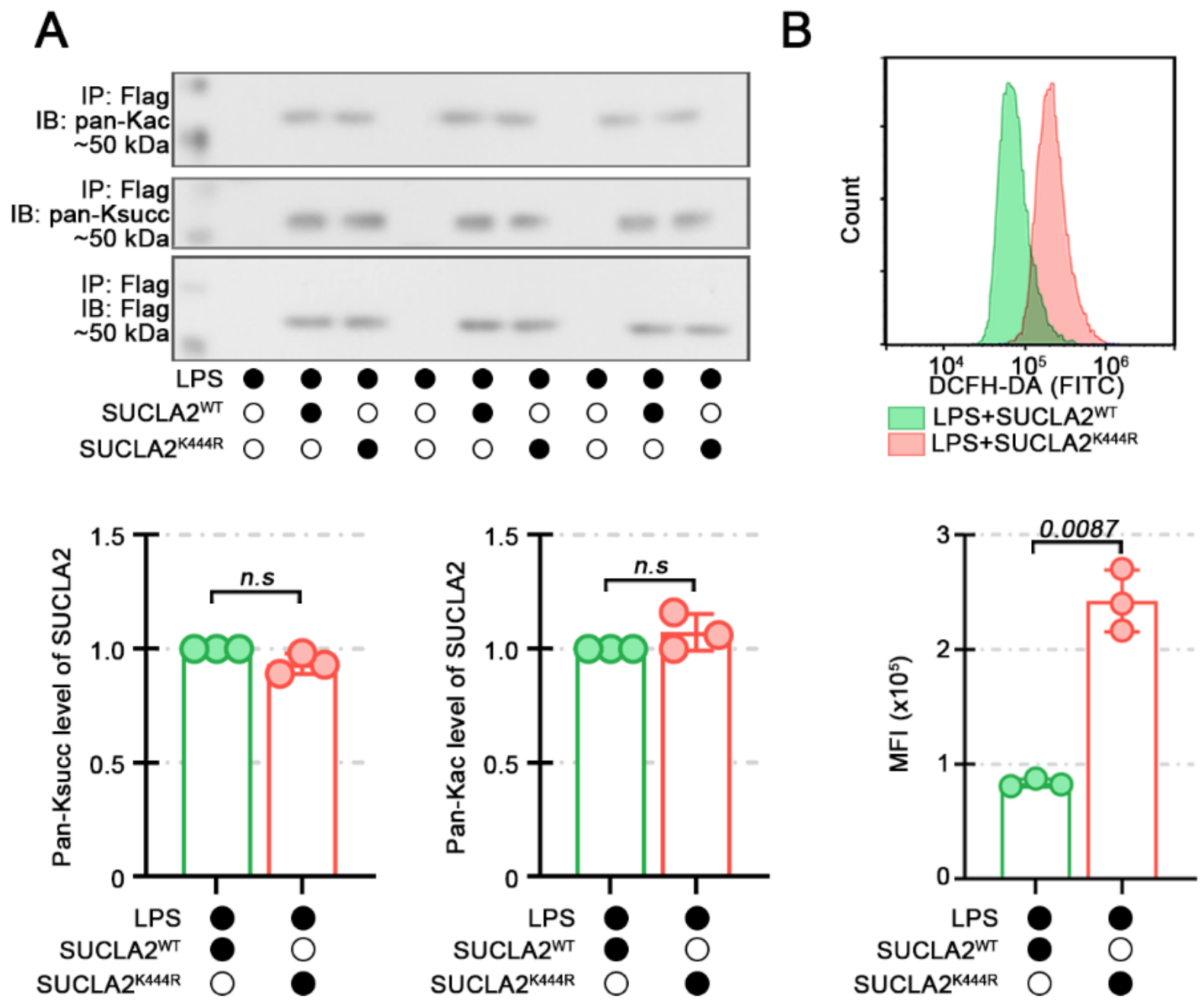

Supplemental Figure 3 Detection of Kac and Ksucc levels in H9c2 cells

(A) Detection of pan-Kac and pan-Ksucc levels in H9c2 cell transfected with SUCLA2^{WT} or SUCLA2^{K444R} plasmid using coimmunoprecipitation assay. Technical replicates: n=3. The statistic comparison methods are Unpaired t test with Welch's correction. (B) Flow cytometry analysis of ROS generation with DCFH-DA probe in H9c2 cardiomyocytes and corresponding MFI values. Technical replicates: n=3. The statistic comparison method is Unpaired t test with Welch's correction. Data are presented as the mean ± SD. SUCLA2^{WT}, wild-type SUCLA2; SUCLA2^{K444R}, arginine mutation at K444 residue of SUCLA2.

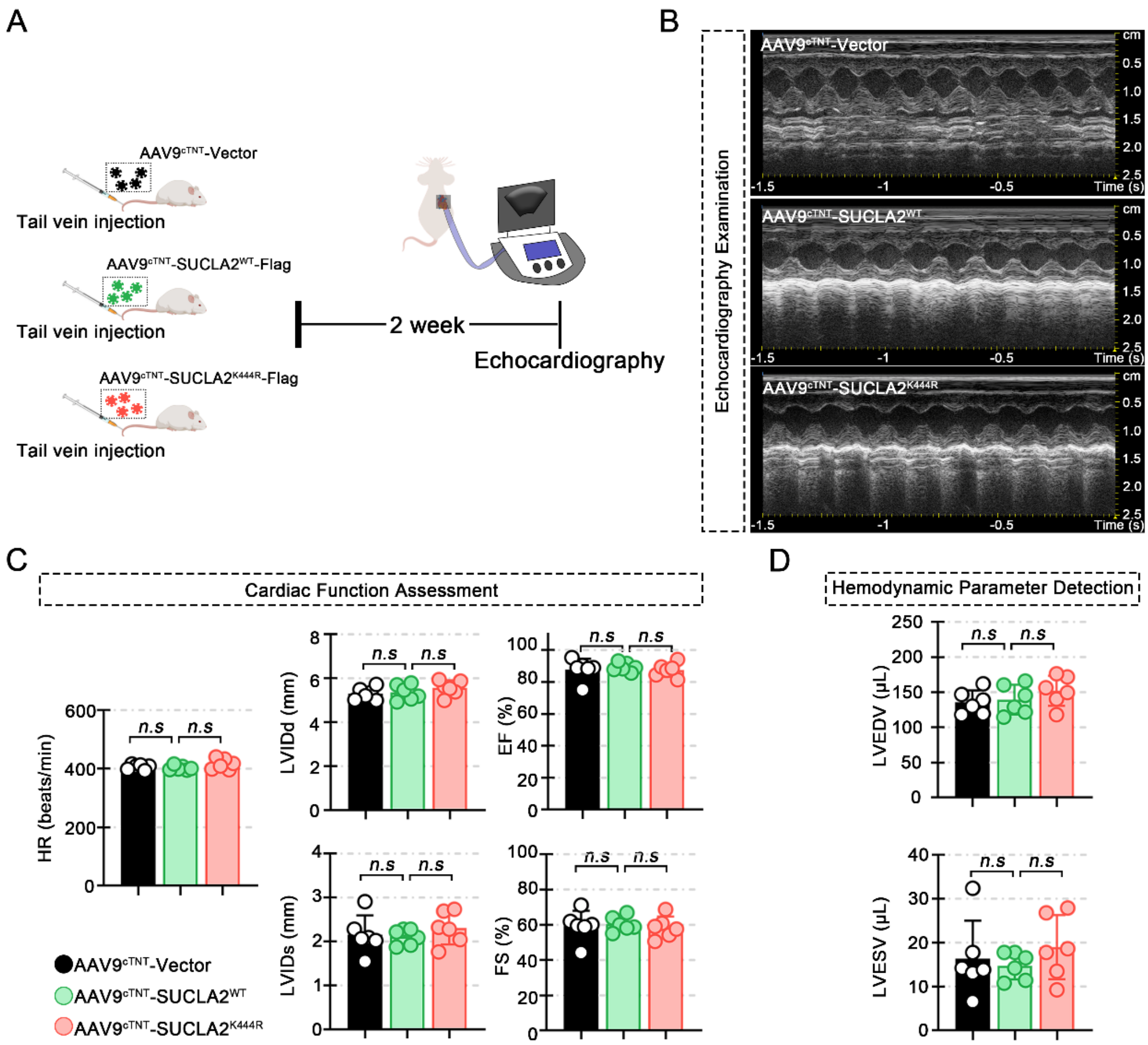

Supplemental Figure 4 Induction of decreased SUCLA2 Kbhb level at K444 site *in vivo*

(A) Schematic diagram of the experimental procedure. Rats were then tail vein injected with wild-type or K444R mutant SUCLA2 adeno-associated virus. After 2 weeks, echocardiography is performed. (B) Representative echocardiographic images. (C) HR, LVIDd, LVIDs, EF, and FS measured by echocardiography. Biological replicates: N=6, The statistic comparison methods are One-way ANOVA with Tukey test. (D) LVEDV and LVESV measured by echocardiography. Biological replicates: N=6, The statistic comparison methods are One-way ANOVA with Tukey test. Data are presented as the mean ± SD. SUCLA2^{WT}, wild-type SUCLA2; SUCLA2^{K444R}, arginine mutation at K444 residue of SUCLA2.

Supplemental Methods

Proteomic and β-hydroxybutyrylomic profiling

Briefly, myocardial tissues from rats in the control and septic groups (n = 4 per group) were homogenized and proteins were extracted using lysis buffer. The proteins were then subjected to tryptic digestion and labeled with 8-plex TMT reagents. Enrichment of β-hydroxybutyrylated peptides was achieved by immunoaffinity purification with anti-Kbhb antibody. The pooled peptides were analyzed by liquid chromatography-tandem mass spectrometry (LC-MS/MS) using an Orbitrap Fusion Lumos mass spectrometer. The processing of MS data was executed through the utilization of MaxQuant, accompanied by the incorporation of the Uniprot Rattus norvegicus database. The identification of Kbhb sites was conducted with a localization probability threshold of >0.75.

Data analysis and quantification

Protein and site quantification was performed using TMT reporter ion intensities. Only proteins with a minimum of two unique peptides and sites with a localization probability of at least 0.75 were considered in the quantification process. The data underwent a process of normalization using median normalization, and missing values were imputed through the utilization of the minimum value approach. Differential expression analysis was performed using Student's t-test with Benjamin-Hochberg false discovery rate (FDR) correction (fold change (FC) >1.2 or <0.83, P <0.05).

Two-stage training strategy of FuncKbhb

We developed functional Kbhb predictor (FuncKbhb), a hybrid deep learning framework, to predict functional Kbhb sites. The details are provided in the Supplemental Methods. The design of FuncKbhb was inspired by widely-adopted strategies for predicting functionally important modification sites [1, 2]. Model training was conducted in two stages. In the first stage, 6,197 Kbhb sites collected from published literature, the CPLM database, and our experimental data were used as positive samples (Table S6), whereas randomly selected unmodified lysine residues were used as negative samples. In the second stage, previously characterized functional Kbhb sites were used as positive samples (Table S7), while the remaining Kbhb sites without reported functional evidence were used as negative samples.

Feature encoding and model development

ESM2-based feature extraction: To this end, the pretrained protein language model ESM2 (facebook/esm2_t30_150M_UR50D) was employed to generate contextualized representations of KSP (25,25) peptides. The self-attention mechanism in ESM2 is mathematically expressed as follows:

$$Attention(Q,K,V) = softmax\left(\frac{QK^T}{\sqrt{d_k}}\right)V$$

Q, K, and V represent query, key, and value matrices respectively, and d_k is the dimension of query vectors. We employed mean-pooling of the final layer outputs to extract 640-dimensional feature vectors. This approach exhibited superior performance in Kbhb classification tasks in comparison to CLS token-based aggregation. LSTM-based feature extraction: We designed a bidirectional LSTM network to capture sequential dependencies in protein sequences. The core LSTM equations are:

$$f_t = \sigma(W_f \cdot [h_{t-1}, x_t] + b_f)$$

$$i_t = \sigma(W_i \cdot [h_{t-1}, x_t] + b_i)$$

$$\tilde{C_t} = \tanh(W_C \cdot [h_{t-1}, x_t] + b_C)$$

$$C_t = f_t \cdot C_{t-1} + i_t \cdot \tilde{C_t}$$

$$o_t = \sigma(W_o \cdot [h_{t-1}, x_t] + b_o)$$

$$h_t = o_t \cdot \tanh(C_t)$$

f_t, i_t, o_t represent forget, input, and output gates respectively, C_t denotes the cell state, and h_t is the hidden state. The final hidden state corresponding to the terminal amino acid residue was used as the comprehensive feature representation.

A composition-based feature framework is employed. The following six established feature encoding schemes were incorporated: ACF: The auto-cross correlation feature captures sequence order and correlation information. AAINDEX: This index contains 566 physicochemical amino acid indices, which reflect various biochemical properties. CKSAAP: The composition of k-spaced amino acid pairs is indicative of residue co-occurrence patterns; GPS: Peptide similarity encoding based on sequence composition; OBC: Position-specific amino acid occurrence features; PseAAC: Pseudo amino acid composition incorporating both sequence and composition information.

Meta-model feature fusion: The eight base models (ESM2, LSTM, and six composition-based models) generated prediction scores for each KSP (25,25) peptide through 5-fold cross-validation. These scores were then concatenated to form an eight-dimensional meta-feature vector:

$$F_{\text{meta}} = [s_1, s_2, \ldots, s_8]^T$$

s_i represents the prediction score from the i-th base model. The meta-model, implemented as a DNN, projected these features into a 128-dimensional space through a fully connected layer with ReLU activation:

$$h_1 = ReLU(W_1 F_{meta} + b_1)$$

Subsequently, the values were concatenated to create an eight-dimensional meta-feature vector:

$$\hat{y} = \sigma(W_3 h_2 + b_3)$$

This hierarchical architecture effectively integrated diverse feature representations while maintaining model interpretability.

Composition-based encoding strategies

The detailed of composition-based encoding strategies included six features: ACF, representing sequence order and correlation information; AAINDEX, containing 566 amino acid indices that reflect various physicochemical properties of amino acids; CKSAAP, indicating the composition of amino acid pairs separated by k other residues; peptide similarity encoded by the GPS method, which assesses the similarity of peptides based on their sequence composition; OBC, representing position-specific amino acids by considering the amino acid occurrence in specific positions of the peptide sequence; PseAAC, denoting the frequencies of amino acids within a peptide, calculated by incorporating both sequence and composition information.

PseAAC

PseAAC is a pseudo-amino acid composition that directly counts the frequencies of 20 typical amino acids in LSP (m,n) peptides. The frequencies of 21 types of pseudo-amino acids in alphabetical order (*A, C, D, ..., Y, **) from LSPs (10,10), and each peptide *i* was encoded into a 21-dimensional vector as follows:

$$V_i = (F_A, F_C, F_D, \cdots, F_Y, F_*)_{21}$$

CKSAAP

CKSAAP represents the composition of amino acid pairs that are separated by *k* other residues within the LSP (m,n) peptides. In this study, all amino acid pairs for $k = 0, 1, \ldots, k_{max}$ were considered, and we encoded each LSP (10,10) *i* into 21 × 21 × (k_{max}+1) dimensional number vectors as follows:

$$V_i = [(C_{AA}, C_{AC}, \cdots, C_{**})_{441}]_{K=0}, [(C_{AA}, C_{AC}, \cdots, C_{**})_{441}]_{K=1}, \cdots, [(C_{AA}, C_{AC}, \cdots, C_{**})_{441}]_{K=K_{max}}$$

For each dataset, the different k_{max} values were evaluated, and the optimal one was determined based on the performance. For our pre-training and fine-tuning datasets, we chose $k_{max} = 0$, resulting in a final CKSAAP feature of 21 × 21 dimensions.

OBC

OBC is the position-specific composition of amino acids[3-7]. In this study, the 21 types of amino acids were alphabetically sorted, and each amino acid was transformed into a binary vector. For the *j*-th amino acid, the *j*-th position was set to 1, and all others were set to 0. For example, “Alanine” was [1, 0, 0, 0, 0, 0, 0, 0, 0, 0, 0, 0, 0, 0, 0, 0, 0, 0, 0, 0] and “Cysteine” was denoted as [0, 1, 0, 0, 0, 0, 0, 0, 0, 0, 0, 0, 0, 0, 0, 0, 0, 0, 0, 0]. For the LSP (10,10), a 420-dimensional vector was generated from the flanking residues.

AAindex

The AAindex is a database that includes 566 well-curated amino acid indices of various physicochemical properties[3, 6, 8, 9]. Each amino acid has a corresponding value for different physicochemical properties. Given physicochemical property h, each LSP (10,10) i was encoded into a 21-dimensional numerical vector:

$$v_i = h_1, h_2, h_3, \cdots, h_{21},$$

h_i is the AAindex physicochemical property for the i-th residue (i = 1, 2, …, 21). For each dataset, 566 physicochemical properties were used individually to train computational models with the penalized logistic regression (PLR) algorithm, and 10-fold cross-validation was performed to calculate the area under the curve (AUC) values. The top 10 most informative physicochemical properties were selected from each dataset based on the highest AUC score to enable rapid training and testing. Finally, each LSP (10, 10) was characterized by a 210-dimensional numerical vector:

$$V_i = v_1, v_2, v_3, \cdots, v_{10}$$

ACF

ACF represents the sequence order information around the PTM site[8]. For each dataset, the top 10 most informative physicochemical properties selected from the AAindex database were used to encode the LSP (10,10). Given physicochemical property h, each LSP (10,10) i was encoded into a 21-dimensional numerical vector:

$$v_i = h_1, h_2, h_3, \cdots, h_{21},$$

h_i is the AA index physicochemical property for the i-th residue (i = 1, 2, …, 21), and the central Kla site was also encoded. Then, the ACF was defined as

$$r_n = \frac{1}{L-n}\sum_{i=1}^{L-n} h_i h_{i+n}\,;\ n = 1, 2, 3, \cdots, m; m = 1, 2, 3, \cdots L-1,$$

L is the length of the LSP (10,10) and equals 21, and m = 1 denotes the correlation of two neighboring amino acid residues. Then, for each LSP (10,10) i, a 20 × 10-dimensional vector V was generated:

$$V_i = [r_1, r_2, r_3, \cdots, r_{20}]_1, [r_1, r_2, r_3, \cdots, r_{20}]_2, \cdots, [r_1, r_2, r_3, \cdots, r_{20}]_i, \cdots, [r_1, r_2, r_3, \cdots, r_{20}]_{10},$$

i represents the i-th AAindex physicochemical property.

GPS

GPS stands for group-based systems, which are used to predict kinase-specific phosphorylation sites in proteins. These systems are based on the hypothesis that similar short peptides exhibit similar biochemical properties for modification. Over the past decade, the GPS algorithm has undergone continuous maintenance and improvement. In this study, we modified the original GPS 3.0 algorithm, and we defined the average similarity score (S) between an LSP (10, 10) P and the positive dataset as follows:

$$S = \frac{1}{N}\sum_{j=1}^{L}(\sum_{i=1}^{N} M[P_j, T_{ij}]) \times W_j,$$

L is the length of the LSP (10,10), L equals 21, and N is the number of positive peptides. T_{ij} is the amino acid at position j of the positive sample T_i (i = 1, 2, 3, …, N). W_j is the weight value of position j, and M represents an amino acid substitution matrixThe BLOSUM62 matrix was used in this study. The weight at each position of the LSP (10,10) was set to 1. The PLR algorithm[10] was used to train models for each dataset and 10-fold cross-validations were performed. The optimal W_j vectors were determined based on the highest AUC value. The average similarity score of amino acid a in the given LSP (10,10) P and residue b in the positive data set was defined as PS_{ab}:

$$S_{ab} = \frac{1}{\sum_{j=1}^{L} C_j}\sum_{j=1}^{L} C_j \times M[a, b] \times W_j,$$

C_j is the number of ab amino acid pairs at the position j. For the 21 types of amino acids, there are 231 unique SAB scores (SAB = SAB). A GPS matrix measures the position-weighted similarity of amino acids between a given LSP (10, 10) and all Kla sites in the positive dataset. Thus, the LSP (10, 10) was characterized as a 231-dimensional vector:

$$V = (S_{AA}, S_{AC}, S_{AD}, \cdots, S_{**})_{231}$$

Multi-feature hybrid system and FuncKbhb models

Base classifiers were built by combining deep neural networks (DNNs) with each encoding scheme. Each DNN architecture consisted of multiple fully connected layers with customized neuron counts, activation functions, and layer normalization. The batch size was set to 32 for the ESM2 and LSTM models and to 2,048 for the six composition-based encodings and the meta-model. The learning rate was fixed at 0.00005 for ESM2 fine-tuning, while other models were trained with a rate of 0.0001.

The fine-tuning of ESM2 involved a progressive learning rate ramp-up from 0.00001 to the optimal value of 0.00005. This was achieved through 150 warm-up steps (5% of the total training steps), followed by cosine annealing decay. The regularization process involved the application of a dropout rate of 0.3 and a weight-decay coefficient of 0.05. During the training process, various settings were investigated, such as feature dimensions, bidirectional variants, and dropout ratios. These settings were thoroughly examined before the final configuration was determined.

Each base model produced prediction scores for KSP (25,25) peptides, optimized via back-propagation under 5-fold cross-validation to ensure robustness and generalization. An 8-dimensional feature vector was constructed as:

$$V_i = (P_{ACF}, P_{AAINDEX}, P_{CKSAAP}, P_{GPS}, P_{OBC}, P_{PseAAC}, P_{ESM}, P_{LSTM})$$

Validation outputs of all base models across folds were then merged with labels to form the

training set of the meta-model.

The meta-model ensemble was implemented as a DNN. Prediction scores from all eight base-models were concatenated to form an 8-dimensional meta-feature vector for each sample. Input features were projected into a 128-dimensional space using a fully connected layer with ReLU activation:

$$ReLU(x) = \begin{cases} x, & x \geq 0 \\ 0, & x < 0 \end{cases}$$

Two subsequent layers (64 and 32 neurons, respectively) reduced dimensionality while retaining discriminative information. The final sigmoid unit mapped the 32-dimensional representation to classification probabilities:

$$P_{ksp}(y) = sigmoid(y) = \frac{1}{1 + e^{-y}}$$

The FuncKbhb predictor used for all subsequent analyses and prioritization was built by retraining the entire pipeline, using the optimal hyperparameters identified during cross-validation. The determined parameters of the FuncKbhb models are listed (Table S9).

Detection of reactive oxygen species (ROS)

Intracellular ROS levels were evaluated by measuring the fluorescence of DCFH-DA using the Reactive Oxygen Species Assay Kit (#BL714A, Biosharp, China).

Biochemical analysis

Superoxide dismutase activity (SOD, #A001) and malondialdehyde (MDA, #A003-1) levels were measured using biochemical analysis kits (Nanjing Jiancheng, China) based on the manufacturer's instructions. Adenosine triphosphatase (ATP) levels were measured using biochemical analysis kits (#S0026, Beyotime, China). Succinyl-CoA synthetase (SCS) levels were measured using biochemical analysis kits (#E-BC-K906-M, Elabscience, China).

Immunofluorescence

Rat myocardial tissue sections were treated with anti-Flag (1:1000; Proteintech, China) and anti-cTNT (1:100; Affinity, China) antibodies overnight at 4°C. Goat Anti-Rabbit IgG H&L (FITC, Abcam, UK) and Goat Anti-Mouse IgG H&L (Cy3®) preabsorbed (Abcam, UK) secondary antibodies were added, followed by 4′,6-diamidino2-phenylindole (DAPI) (Aladdin, China) staining. H9c2 cardiomyocytes were fixed with 4% paraformaldehyde for 15 min at room temperature, permeabilized with 0.1% Triton X-100 for 30 min, and incubated with 1% bovine serum albumin for 15 min. After incubating with primary antibodies against HDAC1 (1:6000; Proteintech, China) and SUCLA2 (1:200; Proteintech, China) at 4°C overnight, the cells were incubated with secondary antibodies, followed by DAPI staining. Immunofluorescent images were captured by fluorescence microscopy (BX53; Olympus, Japan).

Western blot

Rat myocardial tissues and H9c2 cardiomyocytes were lysed using RIPA lysis buffer (Solarbio, China). Total protein was quantified using the bicinchoninic acid (BCA) method. The anti-β-Hydroxybutyryl - Sucla2 (K444) Rabbit pAb was generated by PTMBIO (China). Primary antibodies against SUCLA2 (1:2000, #12627-1-AP, Proteintech, China) and SUCLA2^{K444bhb} (1:100, #CO0915, PTMBIO, China) were used. After washing, the membranes were incubated with an HRP-conjugated secondary antibody. The membranes were further incubated with ECL kit (Solarbio, China).

Mitochondrial membrane potential assay

A commercial JC-1 staining kit (#BL711A; Biosharp, China) was used to measure the mitochondrial membrane potential in H9c2 cardiomyocytes. The cells were suspended in 1 mL of JC-1 working solution, incubated for 20 min at 37°C, and rinsed using the JC-1 buffer. Finally, they were analyzed by fluorescence microscopy.

Co-immunoprecipitation

Rat myocardial tissues or H9c2 cardiomyocytes were homogenized in native lysis buffer (Solarbio, China) supplemented with phenylmethanesulfonyl fluoride. The lysates were quantified using the BCA method and incubated with pre-coated resin containing the corresponding antibodies for 2 h. The proteins bound to the resin were washed and denatured in lysis buffer. The proteins were subjected to SDS-PAGE and Western blot analysis.

Glutathione S-transferase pull-down assay

For the glutathione S-transferase (GST) pull-down assay, pGEX-4T-1 GST vectors were used to construct GST-SUCLA2 expressed in E. coli Rosetta (DE3) Super Competent Cells. The culture was expanded following purification of the obtained GST fusion proteins. His-HDAC1 was incubated with the GST-tagged bait protein at 4°C overnight with rotation. The beads were washed, and the proteins were eluted and detected by Western blot analysis.

References

1 Li WJ, Wang CW, Tao L, Yan YH, Zhang MJ, Liu ZX *et al*. Insulin signaling regulates longevity through protein phosphorylation in Caenorhabditis elegans. *Nat Commun* 2021; 12: 4568.

2 Qin J, Huang X, Gou S, Zhang S, Gou Y, Zhang Q *et al*. Ketogenic diet reshapes cancer metabolism through lysine beta-hydroxybutyrylation. *Nat Metab* 2024; 6: 1505-1528.
